# Diagnosis of invasive fungal infections: challenges and recent developments

**DOI:** 10.1186/s12929-023-00926-2

**Published:** 2023-06-19

**Authors:** Wenjie Fang, Junqi Wu, Mingrong Cheng, Xinlin Zhu, Mingwei Du, Chang Chen, Wanqing Liao, Kangkang Zhi, Weihua Pan

**Affiliations:** 1 Department of Dermatology, Shanghai Key Laboratory of Molecular Medical Mycology, Second Affiliated Hospital of Naval Medical University, Shanghai, 200003 China; 2 Department of Thoracic Surgery, Shanghai Pulmonary Hospital, Tongji University School of Medicine, Shanghai, 200433 China; 3 Shanghai Engineering Research Center of Lung Transplantation, Shanghai, 200433 China; 4 Department of Anorectal Surgery, The Third Affiliated Hospital of Guizhou Medical University, Guizhou, 558000 China; 5 Department of Vascular and Endovascular Surgery, Second Affiliated Hospital of Naval Medical University, Shanghai, 200003 China

**Keywords:** Invasive fungal infections, Fungal diagnostics, Mycology, Detection, PCR, Candidiasis

## Abstract

**Background:**

The global burden of invasive fungal infections (IFIs) has shown an upsurge in recent years due to the higher load of immunocompromised patients suffering from various diseases. The role of early and accurate diagnosis in the aggressive containment of the fungal infection at the initial stages becomes crucial thus, preventing the development of a life-threatening situation. With the changing demands of clinical mycology, the field of fungal diagnostics has evolved and come a long way from traditional methods of microscopy and culturing to more advanced non-culture-based tools. With the advent of more powerful approaches such as novel PCR assays, T2 Candida, microfluidic chip technology, next generation sequencing, new generation biosensors, nanotechnology-based tools, artificial intelligence-based models, the face of fungal diagnostics is constantly changing for the better. All these advances have been reviewed here giving the latest update to our readers in the most orderly flow.

**Main text:**

A detailed literature survey was conducted by the team followed by data collection, pertinent data extraction, in-depth analysis, and composing the various sub-sections and the final review. The review is unique in its kind as it discusses the advances in molecular methods; advances in serology-based methods; advances in biosensor technology; and advances in machine learning-based models, all under one roof. To the best of our knowledge, there has been no review covering all of these fields (especially biosensor technology and machine learning using artificial intelligence) with relevance to invasive fungal infections.

**Conclusion:**

The review will undoubtedly assist in updating the scientific community’s understanding of the most recent advancements that are on the horizon and that may be implemented as adjuncts to the traditional diagnostic algorithms.

## Background

Invasive fungal infections (IFIs) are defined as systemic infections resulting from the establishment of yeasts or molds in deep-seated tissues. In contrast to superficial fungal infections, IFIs are fatal conditions with high rates of morbidity and mortality [1]. The most common invasive infections identified are those brought on by *Candida* species, *Aspergillus* species, *Cryptococcus* species, *Pneumocystis* species, etc. In addition, *Blastomyces*, *Histoplasma*, *Paracoccidioides*, and *Coccidioides* are endemic fungal strains that have also been implicated in causing severe systemic infections in immunocompromised patients [2]. The population at risk for contracting an opportunistic fungal infection includes organ transplant recipients, hematologic patients requiring stem cell transplantation, AIDS patients, diabetics, burn patients, neoplastic disease patients, patients on long-term immunosuppressive therapy, and those with chronic respiratory diseases, among others [3].

Looking at the recent statistics, around 1.9 million patients get an acute invasive fungal infection (IFI) each year, while an estimated 3 million people globally suffer from chronic severe fungal infections. Many of these are life-threatening infections, with an estimated greater than 1.6 million deaths per year attributed to all fungal diseases [4]. Nearly 70% of all IFIs in the world are caused by invasive candidiasis (IC), followed by cryptococcosis (20%) and aspergillosis (10%) [4, 5]. As per CDC’s surveillance data, the in-hospital all-cause (crude) mortality for patients suffering from candidemia is above 25% [6], while invasive aspergillosis (IA), detected in immunocompromised individuals, has an extremely high mortality rate ranging between 40 and 90% [7, 8]. Another added reason of concern is the global emergence of multi-drug resistant fungal species, which worsens the treatment outcomes and enhances the mortality rates. Many fungal species have developed resistance to all four classes of antifungal drugs, i.e. the polyenes, the azoles, the echinocandins and the pyrimidine analogue 5-flucytosine, and a few fungal strains are intrinsically resistant to these antifungal agents, showing high antifungal tolerance [9]. Due to the limited number of antifungal drugs that can be used systemically, treating IFIs is a big clinical challenge.

The aforementioned scenario necessitates prompt and accurate identification of the causal fungi, as speed to diagnosis is the key factor towards improving patient outcome. Although conventional culture tests remain the cornerstone of diagnosing fungal infections, the challenges associated with these tests are manyfold. These include relatively low sensitivity, slow turnaround time, laborious process and the invasive nature of the specimens required for the testing [10, 11]. Blood culture sensitivity for yeasts ranges between 50 and 95 percent, while molds have even lower sensitivity values ranging from 1 to 5 percent [12]. In case of invasive candidiasis, blood culturing is considered a gold standard, but the long turnaround time (in the case of yeasts, up to five days; and moulds, up to four weeks) may put the patient at increased risk due to delay in delineating the necessary treatment plan [13, 14]. In the case of IFIs, studies show significant daily increases in mortality and hospitalisation costs for each day without the proper antifungal medicines, highlighting the severity of such delays [15, 16]. In addition to this, many cryptic fungal species are unable to be isolated and grown on fungal culture media, thus escaping conventional detection. For example, Fontecha and team [17] identified four *Candida* strains (*C. albicans, C. glabrata, C. parapsilosis*, and *C. haemulonii*) which they referred to as cryptic, as they were nonculturable on conventional fungal media and were identified based on molecular methods. Similarly, Arastehfar and team [18] identified nine cryptic species of Candida using one-step multiplex PCR.

In addition to the above shortcomings, the invasive nature of the tissue sample, i.e. biopsies from deep tissues or tissue fluids extracted especially from very old patients, or from neonates, or from patients with hematologic malignancies. Moreover, all the conventional approaches, including microscopy, histopathology, and culture-based tests, rely heavily on personnel with high levels of expertise in fungal identification and detection, and this is practically not possible in every setting. These limitations of conventional tests support the fact that gold standard tests are far from perfection, emphasising the necessity and importance of non-culture methods (e.g., fungal antibody, antigen detection, nucleic acid detection, etc.). So, even though microbiological and histopathological diagnostic tools are still needed for a final diagnosis, newer diagnostic tools with higher specificity and sensitivity may help find and treat IFD earlier which is essential in the clinical setting.

The present reviews aim to highlight the most recent and innovative research in the field of fungal diagnostics. The review initiates with briefly discussing the challenge of antifungal resistance leading to upsurge in fungal infections, Then, the article focuses on the recent advances in: (a) serology-based diagnostics; (b) molecular based diagnostics; (c) biosensor-based assays; and (d) combined new approaches (e.g. use of machine learning, artificial intelligence etc.). Recent advances have been discussed in detail within the relevant clinical context. The imperative need to investigate new technologies that may be able to satisfy requirements in both resource-rich and resource-limited clinical situations is well understood, and this review presents a comprehensive insight into the same. The review will allow the scientific community to be brought up-to-date on the new generation of diagnostic assays necessary to complement the current arsenal of fungal diagnostics.

### Antifungal resistance: an overview

One of the major reasons for early and timely diagnosis of fungal pathogen is the increased mortality rates occurring due to emergence of fungal pathogens not responding to commonly deployed antifungal class of drugs. Fungal pathogens belonging to *Candida, Aspergillus, Cryptococcus*, and *Pneumocystis spp.* have been showing notable rates of antifungal resistance worldwide making antifungal drug resistance a grave concern in both space and time [19, 20]. Of these, *Candida auris* and *Aspergillus fumigatus* are now officially listed on the urgent antimicrobial resistance (AMR) threat list published by the US CDC [21]. As per CDC data, there has been a significant upsurge in clinical cases of *C. auris* (an emerging multidrug resistant yeast) from 329 in 2018 to 1012 in 2021 [22].

Looking at the antifungal agents available, there is a paucity of antifungal drugs unlike antibiotics. Drugs broadly belong to four main classes, including azoles (luconazole, itraconazole, voriconazole, posaconazole, and isavuconazole), polyenes, pyrimidine analogs and echinocandins (caspofungin, micafungin, and anidulafungin). There is a limited choice of drugs already which makes treatment of the infection caused by resistant strain all the more challenging. Briefly discussing on the mode of action of the antifungal classes, we find that triazoles which are the most commonly used antifungals work by targeting specific step (bind to bind to *Erg11* in *Candida* and *Cyp51A* in *Aspergillus species*) in the synthesis of ergosterol, a critical sterol component of the fungal cell membrane [23, 24]. Polyenes such as amphotericin B, bind to ergosterol and this causes membrane destabilization of the fungal cells and eventual cell death. The third class i.e. pyrimidine analogs, such as 5-fluorocytosine (5-FC), are converted to fluorinated pyrimidines metabolites which then destabilize DNA/RNA inhibiting further growth [25]. Echinocandins work by blocking the catalytic subunit of the β-1,3 glucan synthase and thus interfere with β-1,3-d-glucan production, a major cell wall component [23]. Hence, these drugs adopt either a fungicidal or a fungistatic method of action.

The mechanism of acquiring antifungal resistance by fungi is a very vast topic and currently, discussing it in detail is out of the scope of present review. Briefly, discussing the resistance mechanisms adopted by the fungal strain includes (a) decreasing the effective drug concentration, (b) target modification and/or (c) metabolic bypass strategy [26]. The method used for decreasing the effective drug concentration includes the presence of active efflux systems such as the ATP-binding cassette (ABC) transporters and transporters of the major facilitator superfamily (MFS). It has been reported that *C. albicans* contain 28 ABC proteins and 96 potential MFS transporters [27], *A. fumigatus* contains 45 ABC proteins and 275 potential MFS transporters [26] while *Cr. neoformans* contains 29 ABC and 192 MFS transporter proteins, respectively [28]. *C. auris*, an emerging multi-drug resistant yeast also has numerous genes for ABC and MFS proteins conferring azole resistance [29]. Second strategy for decreasing drug concentrations commonly seen is overexpression of drug targets. In this, the fungi tend to overexpress the drug targets and hence more drug is required to attach to them resulting in ineffectiveness of the drug and resistance sets in. For example, *ERG11* upregulation in azole-resistant *C. albicans* and azole resistant *C. tropicalis* [30, 31], upregulation of *Cyp51A* in azole-resistant *A. fumigatus* isolates [32] come under this category. Fungal pathogens also sequester drugs within extracellular compartments as seen with biofilm producing strains of *Candida spp*. wherein biofilm matrix helps in drug sequestration [33] and thus decreases the overall drug concentration.

Drug target alteration is another mechanism commonly reported for azoles (drug target is 14α-lanosterol demethylase) and echinocandins (drug target is β-1,3 glucan synthase) [26]. In case of echinocandin resistance, many clinical isolates of *C. albicans*, *C. glabrata, C. auris, C. tropicalis,* and *C. krusei* have shown target gene modification in two major regions of *FKS1* gene i.e. Hot spot 1 and 2 or HS1 and HS2 regions [34, 35]. Pyrimidine analogues such as 5-flucytosine (5-FC) inhibit DNA and RNA synthesis. *Candida* spp has been reported to escape 5-FC via point mutations in the target gene *FCY1*. Last is metabolic bypass mechanism which is a compensatory mechanism The best example is the loss of function mutations in the gene *ERG3* that codes for a sterol Δ5,6 desaturase. Azole resistant *Candida* strains. This gene product if active can convert 14α-methylated sterols arising from azole exposure into a toxic 3,6-diol product which the fungal cells cannot tolerate. The fungi here divert the toxic effects by loss of function mutation in ERG3 gene and is unable to produce this metabolite resulting in a state of resistance [36, 37]. Apart from widespread use of antifungal drugs in medicine, these drugs are also used widely for plant and crop protection against common plant fungal pathogens in agriculture. Moreover, many opportunistic pathogenic fungi are commonly found within our close living environments. This gives an easy pathway for entry and spread of resistant species into the human chain [38].

Nevertheless, to mitigate the issue of rising drug resistance, the scientific community is into exploring new alternatives which includes phytochemical agents, nanoparticles, herbal extracts etc. [39–42], but the novel drugs reaching the clinical market are still scarce. This scenario emphasises the need for early and accurate diagnosis of fungal pathogen before resistance challenges set in making treatment options limited and all the more difficult. The review now focusses on presenting the detailed discussion on the advances in different diagnostic methods.

### Advances in serology-based diagnostics

Serological testing represents a quicker way of detecting the causal fungi, aiding in the diagnostic decision-making process. These tests are done either to demonstrate antigen or antibody in serum or body fluids of a suspected fungal infection. The advantage of performing serology-based tests is the rapid results obtained, unlike culture methods, and the non-invasive nature of the sample (i.e. blood, urine, sputum, etc.) while acting as a potential prognostic marker [43]. A serology test may give a positive result even if the culture test is negative or the fungal species is nonculturable or the sample is difficult to take from the patient due to some underlying condition [44, 45].

One major limitation of antibody-based testing is seen in immunocompromised or immunosuppressive patients that are unable to elicit adequate levels of antibodies and may show false negative results [46]. However, fungal antigen detection in such patients offers the solution. Polysaccharides or proteins (fungal antigens) secreted during fungus growth may end up in different bodily fluids, making them ideal for detection in both immunocompetent and immunocompromised people as possible disease markers [44]. Still, serology-based testing has its own drawbacks, indicating substantial room for improvement, and the same has been discussed under each subsection.

This assay is based on the detection of (1,3)-β-d-glucan (BDG), a polysaccharide fungal cell wall component. BDG is a pan-fungal antigen present in *Candida* spp., *Pneumocystis jivoveci*, *Aspergillus* spp., *Acremonium* spp., *Fusarium* spp. (exceptions being *Cryptococcus* spp., Mucrorales, and the yeast phase of *Blastomyces dematitidis*) [47]. The only FDA-approved BDG assay is the Fungitell Assay (Associates of Cape Cod, MA, USA). It has been shown to be useful for diagnosing intra-abdominal candidiasis and blood culture negative cases of pneumophila pneumonia [48]. In a meta-analysis study, serum BDG sensitivity and specificity for IC were 75–80% and 60–80%, respectively [48, 49]. In deep seated candidiasis, the sensitivity and specificity were 65% and 75%, respectively [50–52]. The BDG assay is done as colorimetric or in turbidimetric formats and has been included in the EORTC-MSG definition for fungal infection [53]. Till date, there are many BDG assays available besides Fungitell, i.e., Fungitec-G, Beta Glucan-BGStar, Beta-Glucan test (Mauha, Japan) and each assay has varying cutoff values, sensitivity and specificity depending on the fungal strain involved, patient population and assay platform used.

The Fungitell assay has sensitivity and specificity values of 69.9–100% and 73–97.3% for IC and IA, respectively, while the same assay has a sensitivity of 81–93% and a specificity of 77.2–99.5% [54, 55]. The Fungitell assay has been available for two decades as an adjunct test in the diagnosis of IFIs [56]. The test is available in a rapid microtiter plate format with 21 sample batch testing in one go. Although this may be beneficial for serving in large institutions or reference labs that run high sample numbers each day, a low batch format is equally required [57].

With this goal in mind, Fungitell STAT™ was created as an adaptation of the original kit, representing a simple single patient option for checking serum BDG levels in an index value format, allowing patients to be quickly classified as positive, negative, or indeterminate. Like the classical Fungitell assay, the novel format uses Limulus amebocyte lysate (LAL)-based reagents to quantify the rate of para-nitroaniline (pNA) release as a result of hydrolysis by activated BDG sensitive protease zymogens. In a recent study, D'Ordine and team [57] compared the performance characteristics of Fungitell STAT™ and Fungitell on 488 patient samples in terms of linearity of response over the range of Fungitell, positive percent agreement (PPA) and negative percent agreement (NPA) calculation with and without the indeterminate zone, along with the analytical reproducibility (inter and intra-lab variance). PPA and NPA values tell us how many positives and negatives a test identifies that are in agreement with another test used on the same samples. Good linearity was demonstrated with over 250 unique patient samples and lab spiked samples with Fungitell STAT™. The value of PPA with the inclusion of indeterminate was 74% and without indeterminate was 99%, while NPA was 91% with indeterminate value and 98% without indeterminate value. Thus, Fungitell STAT™ has a very strong ability to distinguish between negative and positive in the presence or absence of Fungitell ambiguous samples. Fungitell STAT™ assay represents a good option for running low-batch routine testing with excellent performance, low false positive rates, and high reproducibility.

Another disadvantage of BDG is that it is a non-specific pan-fungal biomarker with low sensitivity values and high false positive results due to cross-reactivity. Racil et al. [58] reported 75% false positive values seen in patients attributed to concurrent bacteraemia, use of haemodialysis or treatment with human immunoglobulin. In 2018, a second commercial assay to detect BDG in plasma samples was introduced. This was the Wako -glucan test (GT) [Fujifilm Wako Pure Chemical Corporation, Osaka, Japan], which was introduced as an alternative to Fungitell in the European market. In a study by Friedrich et al. [59], serum samples were used to compare the performance of the GT assay with Fungitell in patients with IC and *Pneumocystis jivoreci* pneumonia (PJP). The specificity of the GT assay exceeded that of Fungitell for candidemia (98% vs 85%), but the Fungitell assay was higher in sensitivity, i.e. 86.7% as compared to the GT assay (42.5%) for patients with IC and for pneumonia patients, the Fungitell assay showed 100% sensitivity vs 88.9% as seen with GT. However, in a separate study by De Carolis and team [60] in a large cohort study with sera of patients with IA (n = 40), IC (n = 78) and PJP (n = 17) with respect to sera of control patients (n = 187) showed that by lowering the cutoff value when using the Wako test, the sensitivity was improved while specificity remained the same, i.e. 97.3%. By lowering the cutoff to 7.0 pg/mL for the GT, the sensitivity and specificity were 80.0% and 97.3% for IA diagnosis, 98.7% and 97.3% for IC diagnosis, and 94.1% and 97.3% for PJP diagnosis, respectively. Thus, after optimising the GT cutoff value for positivity, the Wako -glucan test performed nearly as well as the Fungitell in a clinical setting. Additional observations by the team were that GT was technically less complex to operate than the Fungitell, simpler to execute and interpret, and that both the single patient option and up to 16 samples in parallel could be run.

Furthermore, Goldstream^®^ Fungus (1–3)-β-d-Glucan Detection Kit (ERA Biology, Tianjin, China) has been widely used in clinical applications for BDG detection, and limulus reagent colorimetry is also used. The diagnostic performance of Goldstream^®^ and Wako for IFD was compared in cases involving *Candida*, *Aspergillus*, and *Pneumospora* infections. Overall, the sensitivity and specificity of Goldstream^®^ for IFD diagnosis was lower than Wako’s (39.6% vs. 43.8%, 83.5% vs. 94.9%) [61]. However, another study using Goldstream^®^ measured serum BDG in 50 patients with PCP, 15 patients with candidiasis, 6 patients with chronic disseminated candidiasis, 15 patients with invasive aspergillosis, 10 patients with mucormycosis, and 40 controls. When the cut off value of Goldstream^®^ was set at 60 pg/mL, the sensitivity and specificity of Goldstream^®^ for the diagnosis of PCP were 86% and 68%, respectively. When the cut off value was set at 31.25 pg/mL, the sensitivity was up to 92%. The specificity, positive predictive value and negative predictive value were 55%, 72% and 85%, respectively [62]. Goldstream^®^ sensitivity was 68%, specificity was 91%, positive predictive value was 66%, and negative predictive value was 91% for paediatric patients with candidiemia [63] (Liu et al. 2015). In another study related to invasive candidiasis in newborns, Goldstream^®^ Fungus (1–3)-β-d-Glucan Detection Kit (Chromogenic Method) had a sensitivity of 76% and specificity of 71.4% [64]. Goldstream^®^ matches IGL-800/IGL-200 (Fully Automatic Kinetic Tube Reader, ERA Biology, Tianjin, China), which is suitable for laboratories with different samples. In 2020, FungiXpert^®^ Fungus (1–3) -beta-d-Glucan Detection Kit (CLIA) (ERA Biology, Tianjin, China) launched, Chemiluminescence immunoassay technology with automatic detection and reducing the detection time to 50 min. Although FungiXpert^®^ Fungus (1–3)-β-d-Glucan Detection Kit (CLIA) has been gradually used clinically, its diagnostic performance for IFD still needs more data support.

Special reference to the detection of dimorphic fungal strains needs to be mentioned. Dimorphic fungal strains refer to the ability of a fungus to generate two types of vegetative cells—either yeast or hyphal in morphology and this switching is modulated by environmental conditions, mainly temperature [65]. There is limited data on the use of Fungitell for serum BDG detection to diagnose endemic mycoses. In one study by Girouard G and team [66], the researchers tested different serum samples from patients with active proven histoplasmosis (*Histoplasma capsulatum*) and blastomycosis (*Blastomyces dermatitidis*). Eight out of the nine sera from patients with culture-confirmed active disseminated histoplasmosis tested positive but the test performed poorly for blastomycosis possibly due to the innate low levels of BDG in *Blastomyces s*pp. The results however suggested that Fungitell can reliably detect BG in cases of disseminated histoplasmosis. But, as reported by Myint et al. [67], cerebrospinal fluid (CSF) BDG was neither sensitivity nor specific to support diagnosis of meningitis caused by *H. capsulatum*. The limited knowledge of the potential role for BDG detection for diagnosis of endemic mycoses emphasizes on additional studies needed in this direction.

*Galactomannan (GM) assay* A useful diagnostic tool is the testing of *Aspergillus* species' cell wall constituents, such as galactomannan. The measurement of cell wall components of *Aspergillus* species, such as galactomannan, is a useful diagnostic tool. GM is the main antigen detected in cases of IA and can be readily detected in bronchioalveolar lavage (BAL) and cerebrospinal fluid (CSF) etc. [68]. This assay’s overall sensitivity and specificity values ranged from 67 to 100 percent and 86 to 100 percent, respectively [69, 70]. GM is specific for *Aspergillus*, unlike the pan-fungal BDG marker, and the majority of medical facilities use GM for routine diagnostics and surveillance of patients at risk for IA. Currently, there is only one FDA-approved assay for the detection of *Aspergillus* GM (Platelia *Aspergillus* enzyme immunoassay (EIA); Bio-Rad, Marnes-la-Coquette, France) in patients’ serum and bronchoalveolar lavage (BAL) specimens. Since then, there have been several attempts to improve or develop new serology-based tests for detecting IA. A mouse monoclonal antibody called JF5 was developed by Thorton et al. [71] that binds to a protein epitope on an extracellular glycoprotein antigen released by *Aspergillus* during its active growth. Based on this, the team developed a lateral flow device (LFD), which will be discussed in a separate section.

Dichtl and colleagues focused on developing a novel JF5-based assay for detecting IA, the galactomannoprotein (GP) ELISA, and compared its performance to the conventional Platelia *Aspergillus* antigen ELISA [72]. The study relied on 267 samples from 49 cases of probable (n = 4) or proven (n = 45) IA. The team observed a strong correlation i.e. R = 0.82 between the measurement results of both tests as determined by Pearson’s correlation. In addition, 156 sera samples (*Aspergillus*-negative control group) were used to determine the specificities of GM and GP. The specificities for the GM (cutoff 0.3) and the GP ELISA (cutoff 0.2) were 96% and 76%, respectively. When using the manufactured cut-off value of 0.5 for GM, the specificity of the GM test was 99% with one false positive case, while for the GP test with a cutoff of 0.4, the specificity was 97% with five false positive cases. Hence, based on the recommended and optimised cutoffs (0.5 for GM and 0.4 for GP analysis), the sensitivity of the GM test and GP test was 40%. In conclusion, the novel GP ELISA was found to be similar to the Platelia GM ELISA in terms of sensitivity and specificity. However, owing to the low sensitivity of the two tests, patients who are at risk of developing IA may require serial testing. The new GP ELISA works just as well as the GM ELISA, so it can be used to diagnose and keep track of IA in high-risk patients. It is also reliable and specific.

The poor reproducibility and the need for repeatability is a drawback observed with Platelia GM-EIA [73]. Gallet and team [74] evaluated the performance of a novel single-sample fluorescent-based EIA assay for detecting *Aspergillus* GM levels in a patient’s sera for diagnosing IA aimed at providing a rapid, easy-to-use robust assay. The team developed a novel single sample test, i.e., VIDAS^®^ GM-EIA (Biomerieux) packaged in a ready-to-use dispensable strip, and tested performance in comparison to Platelia GM-EIA using 126 sera (44 fresh and 82 frozen samples). Comparable diagnostic performance was demonstrated by the area under the curve (AUC) under the ROC curves for the VIDAS^®^GM and Platelia assays (0.892% and 0.894% for the VIDAS^®^GM and Platelia assays, respectively). The ROC curves and best Youden index (i.e., the best balance between sensitivity and specificity) revealed a VIDAS^®^GM cut-off of 0.36, corresponding to a sensitivity of 95.7% and a specificity of 85.7%. The primary benefit of VIDAS^®^GM is that it is a simple, ready-to-use option that provides rapid results (70 min), and this approach can be used to diagnose or routinely screen high-risk populations in a short period of time in order to initiate intervention therapy as soon as possible.

Another improvement has been the release of a novel GM-EIA assay developed by IMMY. In the Bio-Rad GM EIA, a single rat monoclonal antibody referred to as EB-A2 has been used that binds to the fungal galactomannan. The new IMMY GM-EIA assay (just like the IMMY lateral flow kit) involves using two monoclonal antibodies, where one binds to a similar GM epitope as does EB-A2 and the other binds a novel target. In study by White et al. [14], they evaluated the newly released IMMYGM-EIA in a retrospective case–control study. The team discovered that the IMMY GM-EIA displayed a sensitivity of 71% and a specificity of 98%, respectively, with a positive threshold of 0.5. However, by lowering the threshold to 0.27, 90% and 92%, respectively, of the produced sensitivity and specificity were attained. With an observed sample agreement value of 94.7% and a kappa statistic of 0.820, the IMMY and BioRad GM-EIA showed excellent agreement, and the IMMY GM-EIA looks to be a comparable alternative for analysing blood samples. Also, the plate-based design of the assay supports large batch testing with the possibility of automation to further reduce manual error. This advocates further multi-center evaluation and prospective cohort studies to obtain more data on the IMMY-GM-EIA assay and its clinical performance.

*Lateral flow device assay (LFA): new advances* The development of an LFA-based test for the detection of Cryptococcal antigen has been a landmark achievement and has revolutionised the diagnosis of cryptococcal infection, especially in resource-limited settings. *Cryptococcus neoformans* is a dimorphic fungus with its different morphotypes enabling this opportunist pathogen to better adapt and exhibit different levels of pathogenicity in various hosts [75]. In sub-Saharan African populations, cryptococcal meningitis (CM) is a major cause of adult meningitis and accounts for more than 15% of AIDS-related deaths [76, 77]. The cryptococcal antigen, or CrAg, can be found in biological fluids including blood and cerebrospinal fluid, or it can be detected by the traditional culture technique. In addition to these, staining of patient specimens with Indian ink has also been widely used for the diagnosis of *Cryptococcus neoformans* in CSF samples. India ink and 2% chromium mercury were both utilised in a modification procedure that made it possible to clearly identify certain of the organism’s exterior and interior structures [78]. However, India ink, has a low sensitivity that could result in situations of misdiagnosis, which would raise death rates [79]. CrAg can also be detected by latex agglutination tests and enzyme immunoassay (EIA) with more than 99% sensitivity. However, there is still a requirement to use a point-of-care (POC) based test (POC is testing that is performed near or at the site of a patient with the result leading to a possible change in the care of the patient). POC testing is advantageous for rural and distant areas since it can be done without laboratory equipment or facilities [80]. This led to the development of CrAg-LFA. CrAg-LFA is a POC test employing dipstick with monoclonal antibodies that can detect the capsular antigen in the four serotypes (A, B, C, and D) of the pathogenic *Cryptococcus neoformans* species complex and the *C. gattii* species complex. The turn-around time is less than ten minutes, and you don’t need any complicated tools or highly skilled workers. The primary benefit of this test is its capacity to detect extremely low levels of circulating CrAg during the prodromal phase (around 22 days before symptoms), enabling prompt treatment and thus reducing the overall mortality rates [81, 82]. Many companies have been in the development of CrAg-LFA, of which CrAg LFA from Immuno-Mycologics, Inc. (IMMY; Norman, OK) is the only FDA approved one.

**Fig. 1:**
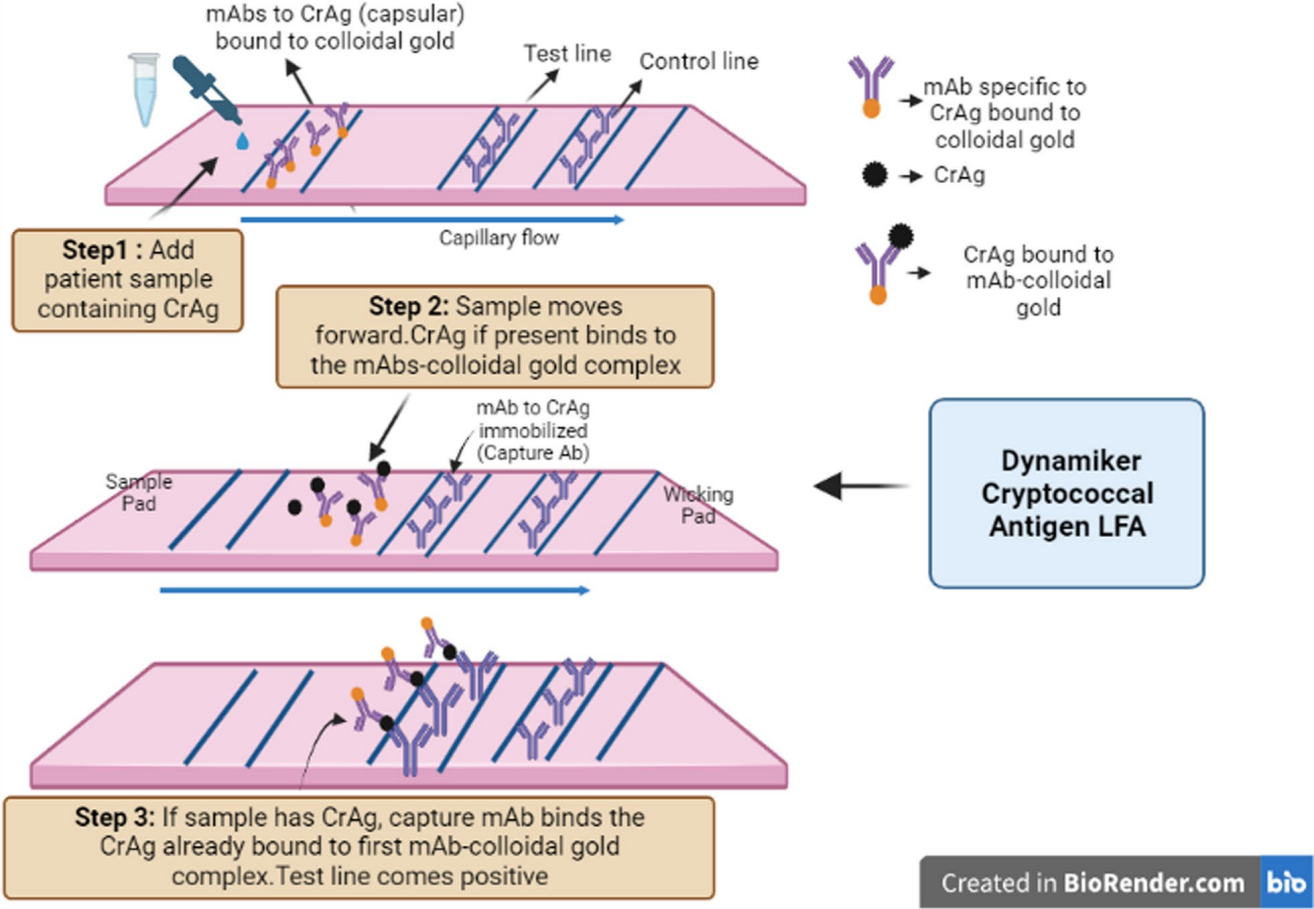
Workflow of Dynamiker Cryptococcal Antigen Lateral Flow Assay (LFA) for detection of capsular polysaccharide antigens of *Cryptococcus* species complex in human serum and cerebrospinal fluid (CSF) [Image created in Biorender.com]

Another dipstick CrAg LFA, called Dynamiker Cryptococcal Antigen LFA, was introduced in 2014 (Tianjin Co., Ltd.). It is a dipstick sandwich immunochromatographic assay for the detection of capsular polysaccharide antigens of *Cryptococcus* species complex (*Cryptococcus neoformans* and *Cryptococcus gattii*) in human serum and cerebral spinal fluid (CSF) as shown in Fig. 1. Kwizera et al. [83] assessed the Dynamiker CrAg-diagnostic LFA's performance in blood, plasma, and CSF samples from symptomatic and asymptomatic HIV patients in comparison to the IMMY CrAg-LFA (reference standard). The researchers examined the effectiveness of the Dynamiker assay using 113 serum samples from individuals with suspected asymptomatic cryptococcal antigenemia and 150 serum, 115 plasma, and 100 cerebrospinal fluid (CSF) samples from HIV patients with symptomatic meningitis. According to the findings, the Dynamiker CrAg LFA has a sensitivity of 98% in serum, 100% in plasma, 100% in CSF from symptomatic patients, and 96% in serum from asymptomatic patients when compared to the IMMY CrAg LFA. The specificity, however, was only 66% in serum from symptomatic patients, 61% in plasma from asymptomatic patients, 91% in CSF from symptomatic patients, and 86% in serum from asymptomatic patients. When Dynamiker CrAg LFA was tested in duplicate, the inter-assay repeatability was 100% across the four sample types, with no observed discrepant results. The Dynamiker test, however, revealed significant levels of false positives, with 11% for serum from symptomatic patients and serum from asymptomatic patients, as well as 14% for plasma from symptomatic patients. In another study by Noguera and team [84], the results were slightly different as for specificity. The team assessed Dynamiker CrAg LFA using 162 cryopreserved serum samples from HIV patients who were asymptomatic and IMMY CrAg-LFA as the reference standard. The team reported strong concordance between the two tests, with sensitivity reported at 100%, reasonable specificity recorded at 89.9%, and accuracy reported at 90.7% for the Dynamiker LFA. Besides this, the Dynamiker CrAg LFA kits are individually packaged, which lowers any potential contamination that may happen when a container is opened several times to retrieve a strip. Nevertheless, these POC alternatives are the need of the hour as they offer a crucial technique to lower the morbidity and mortality of meningeal cryptococcosis, especially in areas where the prevalence of the disease is highest.

It has been reported that the reliability of CrAg LFA falls out recently, thereby hindering an effective treatment of cryptococcal infections and causing a waste of time. Shi et al. [85] evaluated four commercially available LFAs with a set of well-defined *C. gattii/C. neoformans* species complexes. In this study, all seven pathogenic *Cryptococcus* species were detected by the IMMY CrAg LFA and FungiXpert Cryptococcal Capsular Polysaccharide Detection K-Set LFA (FungiXpert, Era Biology, Tianjin, China). However, *Cryptococcus bacillisporus* and some *Cryptococcus tetragattii* strains could not be detected by the Biosynex LFA. This implies the importance of the consideration of the revised cryptococcal taxonomy in the product setup and validation. Furthermore, Liu et al. [86] evaluated the diagnostic performance of FungiXpert LFA and the IMMY CrAg LFA using eight cerebrospinal fluid (CSF) and 119 serum/plasma samples. Compared to IMMY CrAg LFA, the FungiXpert LFA demonstrated 99.1% sensitivity and 98.9% specificity in the qualitative test. The Intraclass Correlation Coefficient of the semi-quantitative results of CrAg titer tests via the two assays was 0.976. This indicates that FungiXpert LFA is also a rapid screening method for the effective and practical diagnosis and treatment of cryptococcosis.

Another major pathogen associated with causing life-threatening fungal infection in immunocompromised patients is *Pneumocystis jirovecii* (PJP) [87, 88]. This fungal strain, formerly called *P. carinii*, is one of the most commonly encountered HIV-associated opportunist infections [89, 90] (Huang et al. 2011; Almaghrabi et al. 2019). Other than HIV patients, patients with underlying malignancies, inflammatory disorders, and autoimmune treatments are also at high risk of being infected with PJP [91, 92]. Pneumocystis cannot easily be cultivated in the laboratory based on the diagnostic assays since it is an extracellular pathogen that is typically located in the alveolar cavity. The gold standard method for diagnosis mostly relies on microscopic cyst detection in respiratory specimens, although it has low sensitivity [93]. This involves the use of expensive and laborious technologies (cytochemical or immunofluorescent staining and/or PCR) applied to respiratory specimens and further use of invasive techniques, such as bronchoscopy, makes the whole process complicated, especially in children or in patients with progressive respiratory insufficiency [94, 95]. These challenges led to the development of a prototype based on LFA offering a simple, rapid, and user-friendly non-invasive technique without involving high-end instrumentation or expertise, allowing a low-cost point-of-care alternative for PJP patients. A gold nanoparticle (AuNP) based LFA for PJP diagnosis has been developed by Tomas and team [96] as a point-of-care diagnostic. In this, the major surface glycoprotein (Msg) and kexin-like serine protease (Kex1) of *P. jirovecii* were synthesised as recombinant synthetic antigens (RSA) and purified. These AuNP-RSA conjugates were then characterised by agarose gel electrophoresis to enhance their ability to interact specifically with serum IgM anti-P antibodies. Finally, these two prototypes—Msg conjugated AuNps and Kex1 conjugated AuNps—were created and examined using pools of sera from individuals with and without PJP. Both immunostrips performed as expected, showing both a test and a control red line with a positive sample and just a control red line with a negative sample. This supports continued development in both resource-rich and resource-poor regions, for quick and simple diagnosis of PJP in patient sera. Avoiding the need for invasive sampling (such as blood or bronchoalveolar lavage has also been a favourable choice. Urine in place of BAL or blood seems a favourable non-invasion sampling option. Marr and his team [97] improved a prototype of a dipstick-style urine-based lateral flow kit for quick and simple IA testing. The immunoassay kit consists of mAb476 conjugated to 40 nm gold nanoparticles dried on a polyester ribbon with results readout in a semiquantitative format as high-positive (++), low-positive (+), or negative. The immunoassay kit is based on a novel galactofuranose-specific anti-*Aspergillus fumigatus* antibody with demonstrated proof of concept in mice and guinea pig models of IA [98]. A cohort of 78 subjects who were being evaluated for suspected IFIs were. Reproducible visual positive was visible in the urine dipstick prototype model at antigen concentrations greater than 0.2 g/mL and beyond. A sensitivity of 80% (95% confidence interval [CI], 61.4–92.3%) was achieved across the entire population when 24 of 30 participants with proved or probable IA had positive dipstick readings. A dipstick reading from two of the 25 controls was also positive, yielding a specificity of 92% (95% CI, 74–99%). Urine LFD demonstrated an estimated sensitivity of 89.5% (95% CI, 66.7–98.7%) and specificity of 90.9% (95% CI, 58.7–99.8%) for individuals with haematological malignancies or other cancers. The promising results clearly indicate the success of adopting this useful and simple to execute assay as a regular screening protocol for the ICU and high-risk patients without the need for any invasive sampling procedure. The dipstick technology requires minimal sample preparation with easy visual interpretation within 30 min with little laboratory skill required. This may be ideal for applying such kits as regular screening tools in resource-limited and rural setups where technical complexity and skilled lab personnel may be a limitation. However, the assay does show cross-reactivity to *Histoplasma capsulatum* and more studies should be conducted to better understand this parameter.

### Advances in molecular-based diagnostic methods

The field of mycology has recently seen many advances in molecular methods for aiding in fungal detection and diagnosis. Molecular methods represent a detection method with fewer variations and a high-performance output, giving more rapid results than culture tests. Moreover, they are the preferred choice for the identification of antifungal drug resistance as well as for the detection of cryptic or non-culturable species. As evidenced by the large commercial assays available for fungal identification (Table 1) fungal PCR tests have been extensively developed, validated, and standardized. From discussing the new PCR assays and related advances, to using newer approaches such as DNA metabarcoding, evolution of new sequencing and bioinformatic tools, the present section will elaborate in a step-wise manner on the recent developments or improvements in molecular-based methods, rendering them more accurate in fungal pathogen identification.

**Table 1:** List of commercially available PCR-based assays for detection of fungi

Commercial PCR and manufacturer	Target species detected	Assay method	Target and specimen used	Assay time	References
Magicplex Sepsis Real eTime Test (Seegne)	*- Aspergillus fumigatus*	Multiplex real time PCR	Unknow; Whole blood	6 h (including DNA extraction)	Camp et al. [142]
*A. fumigatus* Bio-Evolution (Bio-Evolution)	- *Aspergillus fumigatus*	Real time PCR	ITS1 region; BAL	< 80 min after DNA extraction	Denis et al. [143]
MycAssay *Aspergillus* (Myconostica)	Eighteen *Aspergillus* species - *Aspergillus fumigatus* *- Aspergillus flavus* *- Aspergillus terreus* *- Aspergillus niger*	Real-time PCR	18S rDNA; BAL and Serum	4 h	Guniea et al. [144]
AsperGenius® (PathoNostics)	- *Aspergillus fumigatus* *- Aspergillus terreus*	Multiplex real-time PCR	28 S rRNA; BAL, Serum, Plasma, Biopsy tissue	< 3 h	Chong et al. [145]
*Aspergillus* spp. ELITe MGB^®^ Kit (ELITechGroup)	- *Aspergillus niger* - *Aspergillus nidulans* - *Aspergilus terreus* - *Aspergillus flavus* - *Aspergillus versicolor* - *Aspergillus glaucus*	Quantitative real-time PCR	18S rDNA; Bronchial secretions, BAL	Not available (NA)	Grancini et al. [146]
MycoReal *Aspergillus* (Ingenetix)	- *Aspergillus fumigatus Aspergillus flavus* *- Aspergillus nidulans* *- Aspergillus niger* - *Aspergillus terreus*	Real-time PCR	ITS2 region; BAL, Blood, CSF, Tissue	NA	Zeller et al. [147]; Kidd et al. [148]
MycoGENIE® *Aspergillus* Species	*Aspergillus* spp. including: *A. fumigatus*	Quadruplex real-time PCR	28 S rRNA; BAL, serum Biopsy	NA	Dannaoui et al. [149]
Magicplex Sepsis Real eTime Test (Seegne)	*-Aspergillus fumigatus* *- Candida albicans* *- Candida glabrata* *- Candida Krusei* *- Candida parapsilosis* *- Candida tropicalis*	Multiplex real-time PCR	Unknown; Whole blood	6 h	Zboromyrska et al. [150]
FungiPlex Candida (Bruker Daltonics)	*Candida albicans* - *Candida parapsilosis* - *Candida dubliniensis* - *Candida tropicalis* - *Candida glabrata* *- Candida krusei*	Multiplex real-time PCR	Unknown; Whole blood, serum, plasma	< 2 h (after DNA extraction)	Fuchs et al. 2019 [151]
FilmArray Blood Culture Identification (BCID) Panel	*C. albicans,* *C. glabrata,* *C. krusei,* *C. parapsilosis,* *C. tropicalis*	Multiplex real-time PCR assay	Unknown; whole blood	1 h	Salimnia et al. [152]
SeptiFast LightCycler (Roche)	*-Candida albicans* *- Candida tropicalis* *- Candida parapsilosis* *- Candida Krusei* *- Candida glabrata* *- Aspergillus fumigatus*	Multiplex Real-time PCR (DNA melt curve analysis)	ITS region; Whole blood	6–7 h	Steinmann et al. [153]
CandID® and AurisID® (OlmDiagnostics	CandID: - *Candida albicans* *- Candida dubliniensis* *- Candida glabrata* *- Candida krusei* *- Candida parapsilosis* *- Candida tropicalis* AurisID: - *Candida auris*	Multiplex real-time PCR	Unknown; Plasma (CandID) and Blood (AurisID)	45 min (after DNA extraction)	Camp et al. [142]
T2 Candida	*C. albicans, C. tropicalis, C. parapsilosis, C. krusei, and C. glabrata*	PCR along with Magnetic resonance	Unknown; Whole blood	< 5 h	Clancy and Nguyen [100]
PneumoGenius (PathoNostics)	*Pneumocystis jiorovecii*	Real-time PCR	Mitochondrial ribosomal large subunit (rLSU) & dihydropteroate synthase (DHPS) gene mutations; BAL	< 3 h	Prattes et al. [154]
AmpliSens *Pneumocystis* *jirovecii* (carinii)-FRT (AmpliSens)	*Pneumocystis jiorovecii*	Real-time PCR	Mitochondrial large subunit ribosomal(rLSU) RNA gene; BAL, bronchial aspiration, biopsy	130 min (after DNA extraction)	Huh et al. [155]
*Pneumocystis jiorovecii* Bio-Evolution (Bio- Evolution)	*Pneumocystis jiorovecii*	Real-time PCR	Unknown; Bal and Bronchial aspirations	< 80 min	Huh et al. [155]
MucorGenius^®^ (PathoNostics)	-*Rhizopus* spp. - *Mucor* spp. - *Lichtheimia* spp. - *Cunninghamella* spp. - *Rhizomucor* spp.	Real-time PCR	Unknown; BAL, tissue biopsy, serum	< 3 h	Guegan et al. [120]
FungiXpert^®^ PCR (Genobio)	*Crytptococcus neoformans*	Real-time PCR	Unknown; BAL	2 h	Liu et al. [86]

*T2 Candida for rapid diagnosis of candidemia in whole blood* With an estimated death rate ranging from 25 to 40%, candidemia is the fourth most common cause of hospital-associated bloodstream infections [99]. Although blood culturing remains the gold standard, but a blood culture may turn positive in only 50% of cases of candidemia [100]. The classic blood culture performance is far from ideal due to long time of positivity and suppression by antifungal agents. *Candida albicans, Candida tropicalis, and Candida parapsilosis* are usually detected within 36 h, whereas cultures with *Candida glabrata* (a difficult slow-growing organism) can take up to 80 h [101]. A new method has been devised to reduce the time required for invasive candidiasis diagnosis, taking into account the possibility that candidemia can cause sepsis due to a delayed diagnosis and the fact that time is of the essence during sepsis. The US Food and Drug Administration (FDA) approved the qualitative non-culture-based platform T2Candida in 2014 for the diagnosis of candidemia. This test can quickly identify the five most prevalent Candida species from whole blood in around 5 h. Joshi and Shenoy [102] have referred T2 Candida as the game changer diagnosis of invasive fungal infections as they detail out its advantages and its potential in reducing mortality owing to early diagnosis. The test relies on both magnetic resonance as well as molecular methods (i.e. PCR) and being molecular in nature has been included in this section. Briefly, looking into the working of T2 Candida, the process involves: (a) whole blood is collected from patient in presence of EDTA, (b) whole blood tubes are directly inserted into the fully automated T2Dx instrument (T2Biosystems, Inc., Wilmington, MA, USA), (c) T2Dx lyses the Candida cells by mechanical stress, (d) amplification is done using thermostable polymerase and primers for the Candida ribosomal DNA, (d) amplified Candida DNA product is detected using agglomeration of superpara­magnetic nanoparticles bearing target-complementary probes and, (e) nanoparticle clustering causes changes in the T2 relaxation time, that is detected by T2 Magnetic Resonance (T2MR). The resulting product is reported as positive or negative for identification of the 5 common Candida species (*C. albicans, C. tropicalis, C. parapsilosis, C. krusei*, and *C. glabrata*) which account for > 95% of candidemia cases [103–105]. The test can be done with just 2–4 mL of whole blood, reason why it can be used in paediatric too. The mean turn-around-time is < 5 h and the limit of detection is as low as 1–3 CFU/mL of whole blood compared to 100–1000 CFU/mL typically required for conventional PCR-based methods. The overall sensitivity and specificity of T2 Candida is 91.1% and 99.4% respectively and a NPV of 99.4% as per a multi-center trial given a prevalence of candidemia of 5% in a general hospital/ICU setting [106]. These outstanding parameters definably make T2 Candida a game changer thus speeding up the start of antifungal therapy before the picture turns ugly for both patient and the physician. Further, T2MR not only covers the five Candida species but also is able to detect is able to rapidly detect six common bacteria (so-called “ESKAPE” pathogens including *Escherichia coli*, *Staphylococcus aureus*, *Klebsiella pneumoniae*, *Acinetobacter baumanii*, *Pseudomonas aeruginosa*, and *Enterococcus faecium*) [107].

Bilir et al. [108] estimated that T2 candida panel has a huge economic impact by employing 1-year decision-tree model. The model calculated the potential savings per patient with candidemia in a hospital with 5100 yearly high-risk patients to be $26,887, or a 48.8% reduction in hospital expenses. While avoiding 60.6% of mortality brought on by candidemia. Additionally, the rapid Candida identification showed the potential to save over 30 lives annually in a typical hospital setting, which translates to a mortality reduction of 60.6%. A major advancement has been the development of the new T2 C.auris panel by T2 biosystems. *C. auris* has been recognized by the CDC as a serious global health threat because of being multi-drug resistant to major classes of antifungal drugs. When compared to culture methods, which required 14 days, the T2Cauris panel showed considerable time advantages (5 h) and the inability to detect low amounts of *C. auris*. When compared to existing molecular diagnostic tests for *C. auris*, the T2Cauris panel has a greater than 100-fold increase in sensitivity and can detect levels as low as 5 CFU/mL [109, 110].

Needless to say, T2-MR is definitely a breakthrough technology for the detection of candidemia with significant impacts on patients’ mortality and morbidity rates, hospital stays and hospital costs. Given its excellent performance parameters, T2Candida is highly advocated to be incorporated into diagnostic algorithms and guidelines in conjunction with blood cultures to guide management of patients with suspected invasive candidiasis especially in ICU settings or other high prevalence settings. Additionally, the high NPV enables clinicians to confidently halt or de-escalate antifungal medication so as to start other treatment therapies well in time. Thus, positive T2MR results need to be evaluated in light of the anticipated disease prevalence in the particular clinical scenario. Whether T2Candida can be used as a monitoring tool for assuring complete clearance of candidemia could be the subject of more research as past study has shown that T2Candida can remain positive even after blood cultures are clear [106].

The deep-seated infections originate either by hematogenous seeding or due to non-hematogenous introduction of *Candida* into sterile sites, most commonly the abdominal cavity following GI tract disruption or via an infected peritoneal catheter [100]. However, blood cultures may be unable to detect candida and give negative results. This may be due to either the concentrations of viable candida cells are insufficient to be detected within a collected sample, or there is intermittent or transient release into the bloodstream and culturing timings have not matched [111]. T2 Candida has been shown to give promising results in detecting deep-seated invasive candidiasis (IC) in patients whose blood cultures were negative and later confirmed positive by tissue biopsy [104]. Still, more studies are needed to determine the performance of T2MR in diagnosing invasive candidiasis without candidemia.

*Advances in PCR assays in fungal diagnostics* PCR was the first nucleic acid amplification method to be developed. Since then, new and advanced PCR variations, including nested PCR, real-time PCR, multiplex PCR, etc., have been created. The platform for mycological testing and identification has benefited from improvements in PCR-based techniques. Fungus-specific primers for PCR and quantitative real-time PCR amplification has been used for diagnosis of *Aspergillus*, Candida, Mucorales, and *Pneumocystis jirovecii* infections [112]. A PCR assay for the detection of fungal nucleic acids may be the best diagnostic strategy because (a) more sensitive than current culture-based methods, (b) comparatively less time consuming than culture tests, (c) being applied to many clinical sample types (blood, body fluids, BAL, CSF etc.) and, (d) applied for detection of nonculturable species or when culture tests are negative due to early start of antifungals. The readers need to know that discussing all the advances made in various variants of PCR is not possible, hence we will emphasizing the most recent developments (2015 onwards) related to diagnosis of invasive fungal pathogens in this section.

*Multiplex PCR advances* The concept of multiplex PCR (m-PCR) is not new. With the objective of overcoming the inherent issues of high cost and to further improve the diagnostic capacity of PCR, a variant called multiplex PCR was introduced. A m-PCR allows the simultaneous detection of multiple targets in a single reaction well, with a different pair of primers for each target. This saves on the cost, time, efforts but with no compromise of test utility. A real-time multiplex PCR may concurrently detect between two and five pathogenic species using species-specific primers and probes tagged with various fluorescent dyes for each target species [113–115]. Additionally, m-PCR can distinguish between extremely closely related organisms with enough specificity to detect multiple pathogens, which significantly lowers expenses. There are a large number of commercial m-PCR kits available in market for detection of the common fungal pathogens (Table 1), but few commercial assays are worth mentioning We have kits e.g. SeptiFast (Roche Diagnostics) and MycAssay *Aspergillus* that do not require prior fungal culture and DNA amplification is performed directly from clinical samples saving an additional step. SeptiFast m-PCR uses a modified DNA extraction protocol that enables to yield a higher sensitivity (90.5%) detecting pathogen in as low as 100 μL blood volumes thus allowing use of the kit in cases of diagnosis of fungal neonatal sepsis wherein obtaining higher blood volumes from neonates and preterm is a limitation [116]. Another PCR advancement is the AsperGenius (PathoNostics, Maastricht, the Netherlands), a unique multiplex real-time PCR assay consisting of two multiplex real-time PCRs, one to identify the clinical *Aspergillus* species in the sample, and second m-PCR to detect the mutation in the CYP51A gene of A. fumigatus that causes azole resistance. Thus, AsperGenius is actually a genius approach simultaneously detecting the causative agent and finding the presence of drug resistance directing from the patient’s BAL sample. The kit showed an overall sensitivity, specificity, PPV, and NPV are 84.2%, 91.4%, 76.2%, and 94.6%, respectively [117]. Next, we have the FilmArray Meningitis/Encephalitis (ME) panel (BioFire Diagnostics, Salt Lake City, UT), the first m-received FDA approval in Oct 2015. FilmArray Meningitis/Encephalitis panel detects one fungal target, *Cryptococcus neoformans/Cryptococcus gattii*, in addition to bacterial and viral targets in CSF [118]. Of interest, is the new commercial PCR assay for detecting invasive mucormycosis (IMM) i.e. MucorGenius (PathoNostics). The kit is based designed to detect the 28S multi-copy gene in the most prevalent clinically relevant species: pan-Mucorales DNA, *Rhizopus* spp., *Mucor* spp., *Lichtheimia* spp., *Cunninghamella* spp., and *Rhizomucor* spp. The kit allows direct detection in BAL samples and rapid result in less than 3 h [119, 120]. The assay was also evaluated for detecting IMM in serum or tissue samples [119] and results showed 91% sensitivity in IMM tissue samples. Also, mucorales DNA was detected in serum of patients with probable/proven IMM (100%) and in 29% of the possible cases. In another multicenter retrospective study [121], MucorGenius was tested on 106 blood samples from 16 patients with culture-positive invasive mucormycosis and found an overall sensitivity of 75%. The positive results by the kit preceded a positive culture by a mean duration of 81 days indicating that Mucorales DNA can be detected in patients with suspected IMM much earlier and at initial stages of infection (unlike traditional tests) giving enough time to have a better control on resolving the fungal infection from the host system. One specific advantage of the MucorGenius^®^ assay is that it can be run in parallel with an *Aspergillus* specific assay by the same manufacturer (AsperGenius^®^). Hence, in a single run, the BAL sample can be testing for presence of both molds simultaneously using four different detection channels (green, yellow, orange, and red for AsperGenius^®^, and yellow and red for MucorGenius^®^). This approach could be very relevant in a clinical setting detecting coinfections with both molds guiding the optimal treatment therapy. Coinfections or mixed infections with multiple molds are a significant reason for suboptimal treatment outcomes. Cases of mixed infection have been highlighted in immunocompromised patient SARS-CoV-2 [122, 123]. This requires high degree of precision and implementation of accurate diagnostic assays covering larger panel of suspected fungal strains. With this objective Carvalho-Pereira and team [124] developed novel multiplex PCR consisting of two panels i.e. Candida Panel (to identify *C. albicans, C. parapsilosis, C. glabrata, C. krusei, C. tropicalis*), and the Filamentous Fungi Panel (to identify *A. fumigatus, A. flavus, A. terreus, A. niger* and *R. arrhizus*) using species specific primers. This is one of its unique kind of m-PCR designed covering the identification of the ten most clinically relevant fungal species causing invasive diseases from positive blood culture as well from tissues specimens from biopsy or from sterile sites etc. Further, the novelty of this approach lies in the fact that the assay uses species specific primers outside the mitochondrial or ribosomal DNA, which reduces cross-amplification from non-target species. The assay showed no cross-reactivity with nontargeted species and exhibited a limit of detection of 10 to 1 pg of DNA and showed promising detection of fungal DNA from spiked human serum with no interference from human DNA. Further, this dual panel m-PCR used easy visualization of final results which is based on presence of correct size fragment and the specific fluorescent color, ruling-out unspecific amplifications. The customization of the assay to widen the panel to include new species as per the epidemiology of the specific geographical region can be a future possibility.

Moving ahead, in light of emerging resistance to fungal drugs, simultaneous detection of the clinical fungal species along with detection of fungal resistance would be an added advantage within the same run, time and resources. With this objective, a dual panel multiplex PCR assay was designed in order to detect the major fungal species causing invasive infections and also to identify resistant species. Genus specific primers for *Candida, Aspergillus* and *Fusarium* spp. isolates and species-specific primers for *C. glabrata, C. krusei* and *A. terreus* were designed and optimised for multiplex detection of the fungal targets. While the species-specific assay identified 10 pg–1 ng DNA, the genus-specific multiplex PCR assay had a detection limit of 0.1–1 ng DNA [125]. Such dual panel PCR versions would permit speedy and reliable differentiation between resistant species as well as the detection of clinically significant fungi, aiding in the early implementation of an antifungal regimen.

In another novel approach, a multiplex real-time quantitative PCR detecting system was optimised for rapid diagnosis of *C. auris*, an emerging multidrug opportunistic pathogen directly from spiked serum samples [18]. The m-PCR exhibited high analytical specificity and sensitivity i.e. 100% specificity and sensitivity of up to ten genomes of *C. auris* with good reproducibility. As *C. auris* continues to present itself as a multidrug-resistant opportunistic yeast in clinical settings, this novel m-PCR hold the potential as a promising approach due to its ability to directly detect *C. auris* and closely related species from serum samples of suspected patients.

*Droplet PCR* Digital droplet PCR (ddPCR) is a relatively new form of PCR with a wide range of applications. The principle relies on nucleic acid amplification as a normal PCR but the distinctive feature is that a droplet PCR separates the reaction mixture into hundreds to millions of partitions and detects the amplification in real time or endpoint. The target sample is massively partitioned into twenty thousand nanoliter-sized droplets using the template nucleic acid. These droplets contain target sequences. Every droplet is a PCR sample instead of one. After amplification, droplets are tested to see if they contain the desired sequence (positive droplets) (negative droplets). The fraction of positive droplets determines the template concentration in the original sample using a Poisson distribution [126, 127]. Unlike qPCR, ddPCR does not require extrapolation, standard curves, or references samples. The absolute quantification achieved at the end of the amplification, after the experiment is finished, is the basis of this approach. Additionally, ddPCR is less sensitive than qPCR to primer-template mismatch, PCR inhibitor presence (many clinical samples do contain inhibitors), and varied amplification efficiency [128]. The amount of samples and reagents needed is modest, the reaction volumes in ddPCR are in the nano- and picoliter range, and this further reduces the overall cost of running a ddPCR. These advantages of droplet PCR have been exploited for absolute detection and quantification of fungal pathogens as well. DD-PCR has been used to detect *A. fumigatus* and *A. terreus* in respiratory airway specimens. The team compared in a head-to-head fashion the qPCR with the ddPCR technique by testing both in 20 sputum specimens with known *Aspergillus* status. ddPCR was superior for the detection of *A. terreus* particularly at very low DNA abundance with greater resistance to PCR inhibitions compared to qPCR. Chen and co-workers [129] also found superior results when applying ddPCR for detection of candida DNA in whole blood as compared to qPCR. This new technique was able to detect as low as 4.5 DNA copies per reaction in blood samples with high specificity and good reproducibility. ddPCR also showed higher sensitivity of 94% vs. 69% for culture and 79% for qPCR method thus advocating its application in early candida diagnosis with minimal blood volumes required. The ddPCR has also been tested for its efficacy in neonatal invasive fungal infections [130]. The fatality rate due to neonatal IFIs in the neonatal ICU can go as high as 36% [131].Further, an IFI progresses rapidly that making early and accurate diagnosis so very crucial. Based on highly conserved 18S rRNA gene sequence of fungi, ddPCR detection system was established through primer design and system optimization. The study was done on 83 neonatal patients with high-risk factors and/or clinical symptoms of IFI. ddPCR exhibited specificity of 100% and high sensitivity detecting upto 3.2 copies/μL of test blood with good repeatability. Further, due to the very minimal blood volumes required in ddPCR, the technique is highlight apt for neonatal patients especially preterm infants.

*Combined PCR approaches* This subsection is devoted to the developments wherein PCR has been combined with techniques go obtain high order precision and accuracy. Sepsis flow Chip platform and ePlex are two such approaches worth mentioning. Sepsis Flow Chip (Master Diagnostica, Spain) is a platform for the detection of the most prevalent pathogens in systemic infections from positive blood cultures. It combines multiplex PCR with a reverse dot blot hybridization. The European Economic Area has certified the DNA microarray-based assay Sepsis flow as a suitable in vitro diagnostic tool. The working involves multiplex PCR amplification with biotinylated primers, followed by an automatic reverse hybridization in membrane containing particular probes for detecting the most significant bloodstream infection pathogens and the most significant genetic resistance determinants in microbes. It is designed for simultaneous detection of 40 pathogens including resistance strains of bacteria and among yeasts, Candida species. This diagnostic assay enables the rapid detection of bloodstream infection-causing microbes and their key antibiotic resistance indicators directly from positive blood cultures in three hours turn-around time. The test exhibited high values of sensitivity (93%) and specificity (100%) regarding Candida species, respectively [132].

Next, the GenMark Diagnosis, USA-based ePlex^®^ system is a completely automated platform for the analysis of positive blood cultures combing microfluidics, PCR and electrochemical detection techniques all in one. This FDA cleared assay is one of its kind covering a total of 16 fungal species/genera simultaneously (BCID-FP panel) in addition to bacterial pathogens and related resistance markers. This large panel of fungal pathogens includes: Candida species (*C. albicans, C. auris, C. dubliniensis, C. famata, C. glabrata, C. guilliermondii, C. kefyr, C. lusitaniae, C. parapsilosis, C. tropicalis C. krusei*), *Cryptococcus neoformans*, *C. gattii*, *Fusarium* spp, and *Rhodotorula* spp [133]. ePlex one-step single-use cartridge assay relies on a different sample preparation step i.e. utilising microfluidic phenomenon of electrowetting technology followed by multiplexed nucleic acid extraction, amplification and digestion. In electrowetting, discrete droplets on the surface of a printed circuit board with a hydrophobic coating are directly manipulated by electrical fields which enables rapid thermal cycling [134]. The final target is however detected using the proprietary method of electrochemical nucleic acid detection called eSensor technology. eSensor technology is based on the principle of competitive DNA hybridization and electrochemical detection. Briefly, eSensor technology recruit’s ferrocene derivatives to the surface of gold-plated electrodes via an oligonucleotide “sandwich” method. Following PCR thermocycling, single-stranded amplicons are produced via exonuclease digestion, which are then annealed to signal probes that are conjugated to ferrocene. The capture probes are bound to the gold electrodes and the signal probes maintain an amplicon-overhang that is complimentary to the capture probes. Now, the patient DNA is mixed with the signal probe solution and if the target DNA is present, rapid hybridization to the signal probe occurs. The solution is then pumped through the cartridge chamber and the target DNA/signal probes start competing with the pre-assembled capture probes on the gold electrodes. With this, there occurs changes in the iron-redox cycling at each electrode and these changes are detected by electrochemical detection software giving the presence or absence of target DNA that we are looking for in the patient [135, 136]. Since the eSensor technology is highly specific for its target DNA, this test is less prone to sample contamination risk and do not require time-consuming washing steps. Furthermore, the requirement for repeated oligonucleotide annealing to produce an electrochemical signal enhances the overall diagnostic specificity. Rapid diagnostic turnaround is made possible by the speed at which amplicons are recognised and the short time needed to sweep a voltage across individual electrodes. ePlex shows a specificity of 100% and a sensitivity ranging from 99.8 to 100% for fungal pathogens [137]. While testing on 105 clinical samples, the PPA was 92.4%, and the NPA was 99.9% [138]. The panel can easily distinguish between contaminants and real infections more quickly, allowing for rapid de-escalation and discharge of patients with bloodstream infections 2–3 days earlier than with traditional approaches [133]. Combining blood culture and ePlex could shorten the turnaround time for detecting sepsis fungi from 72–96 to 10 h [139], and this can be an added value for clinical management of patients with bloodstream infections and possible sepsis well before time.

**Fig. 2:**
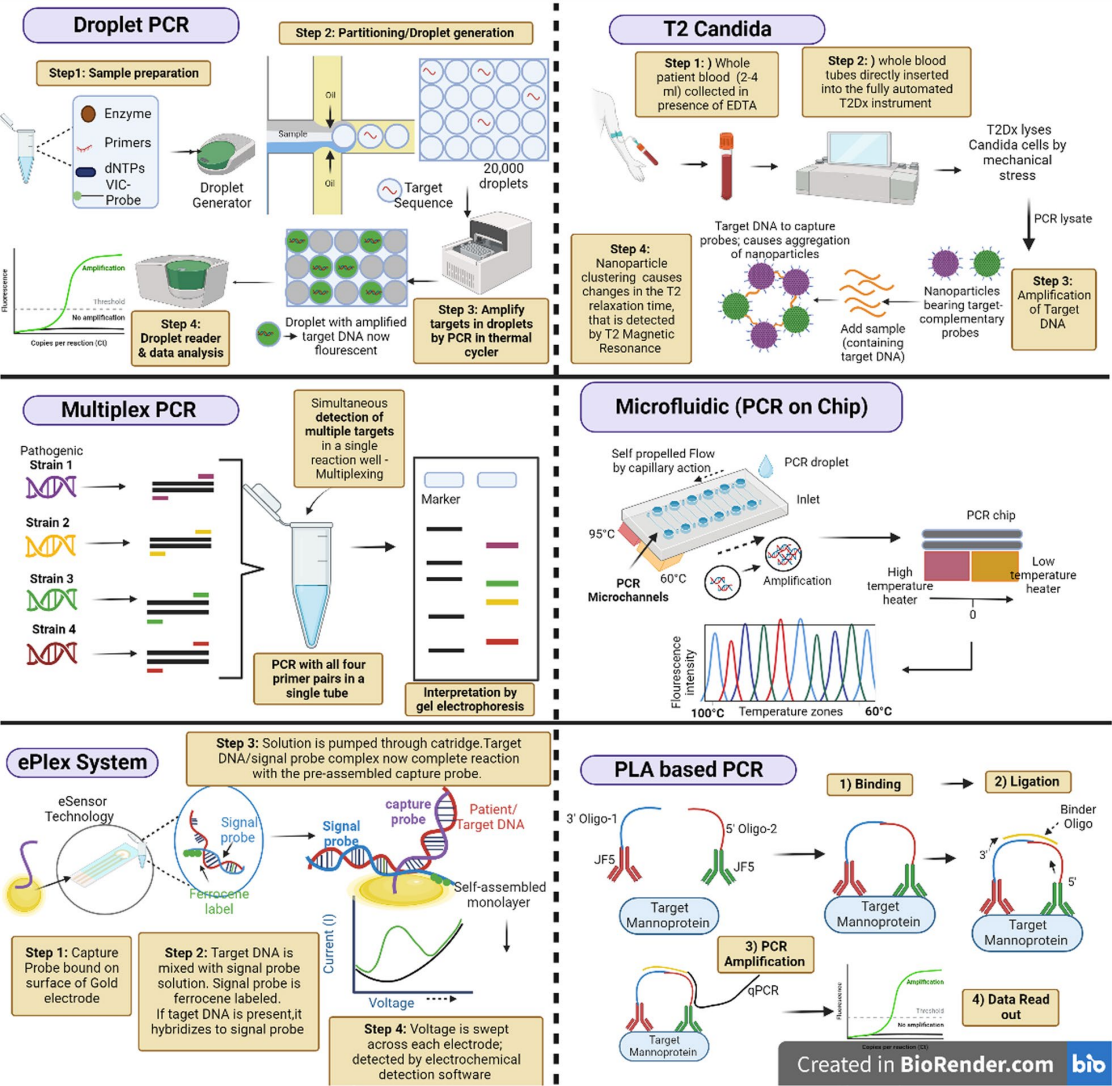
Workflow of various PCR based advanced technologies that are of potential use in the field of diagnostics (PLA: Proximity ligation assay; VIC: florophore). [The figure has been created in Biorender.com]

Of interest, another combined approach is the proximity ligation assay (PLA) for early detection of invasive aspergillosis (IA). PLA combines the specificity of antibody-antigen recognition with the sensitivity of real-time PCR (qPCR) detection. Briefly, the working principle involves utilizing two biotinylated proximity probes based on the *Aspergillus* specific monoclonal antibody JF5 targeting antigenic mannoproteins. These two monoclonal antibodies conjugated to non-complementary oligonucleotides, are added to the test sample. Next, when the two antibodies bind to epitopes in proximity of each other only then, a binder oligonucleotide hybridize with both strands thus creating a single DNA strand that may be amplified by PCR and identified using qPCR [140]. No cross-reactivity was seen between the soluble antigens from the *Candida*, *Mucor*, *Fusarium*, *Rhizopus*, *Lichtheimia*, or *Cryptococcus* species, indicating that the assay was very specific. In spiked serum and saline samples, PLAability^®^’s to detect the *Aspergillus* target protein was established, with sensitivity 10 × to 100 × higher than the GM assay and better sensitivity than the LFD, which uses the same Mab [141]. This has important implications for early diagnosis and targeted treatment of IA. The workflow of various PCR based advances discussed above in this section has been diagrammatically detailed in Fig. 2.

*DNA metabarcoding* DNA barcoding is a well-known method of species identification that uses a short sequence of DNA from a specific gene or genes. The fundamental concept behind DNA barcoding is that by comparing each individual sequence to a reference library of these DNA segments, commonly referred to as “sequences,” it is possible to specifically match an organism at species level. Metabarcoding is an extension of barcoding that enables species to be identified from mixed samples using high-throughput sequencing techniques [156, 157]. Gene regions can serve as biological barcodes for various organisms. The “Barcoding Gap,” or the difference between intraspecific (within species) and interspecific (between species) variation, is why these gene areas were selected [158, 159]. Nuclear DNA’s internal transcribed spacer (ITS), which has various copy numbers, offers the best species-level precision and allows for the creation of both fungal and universal primer [160, 161]. Thus, ITS1 and ITS2 sub-regions have been applied as metabarcoding markers [160, 162]. Completeness of reference sequences is crucial for the successful implementation of DNA barcoding/metabarcoding, and curated databases like UNITE, MaarjAM, ISHAM DNA barcoding, and NCBI RefSeq play a significant role [159, 160, 163].

However, a significant shortcoming of the ITS region includes its inability to distinguish between phylogenetically related species with potentially identical or hardly distinguishable sequences [164, 165]. Only about 75% of all fungal species can be reliably identified to species level by the ITS1/2 genetic locus. Translocation elongation factor 1 alpha (TEF1) gene has been proposed as the secondary fungal DNA barcode. The ability to create universal primers like EF1-1018F and EF1-1002F and its high species discrimination across fungal taxa led to the selection of the translational elongation factor 1 (TEF1) [166]. The ISHAM Barcoding Database has also been expanded to include sequences for both barcoding regions, making it possible to practically implement the dual barcoding scheme into clinical practise in order to accommodate this secondary fungal DNA barcode [167]. The dual fungal DNA barcoding scheme is the result of combining both loci (ITS and TEF) which led to generation of 270 new secondary fungal barcode sequences. When the nucleotide diversity of 43 different species of fungi was analysed, it was discovered that the TEF region is less diverse than the ITS region and that the intraspecies variation of the TEF1 gene is typically less than 1.5%, making it a more discriminating marker. Dual systems provide better species identification than using a single barcode alone since the combination of the two barcodes considerably improves the discriminatory power, enabling more precise identification. The team also proposed a metabarcoding scheme using both types in a diagnostic setting. After receiving a clinical sample followed by initial assessment based on its morphologic and/or biochemical characteristics, the sample is subjected to primary fungal DNA barcoding (ITS1/2 region); if the sequence shows less than 98.5% identity to a given ITS reference sequence in the database, secondary fungal DNA barcoding (TEF1) is performed. Thus, the discussion above strongly advocates the use of the dual fungal DNA barcoding scheme for identification of fungal pathogens and its implementation into routine diagnostics.

### Next generation sequencing (NGS)

**Fig. 3:**
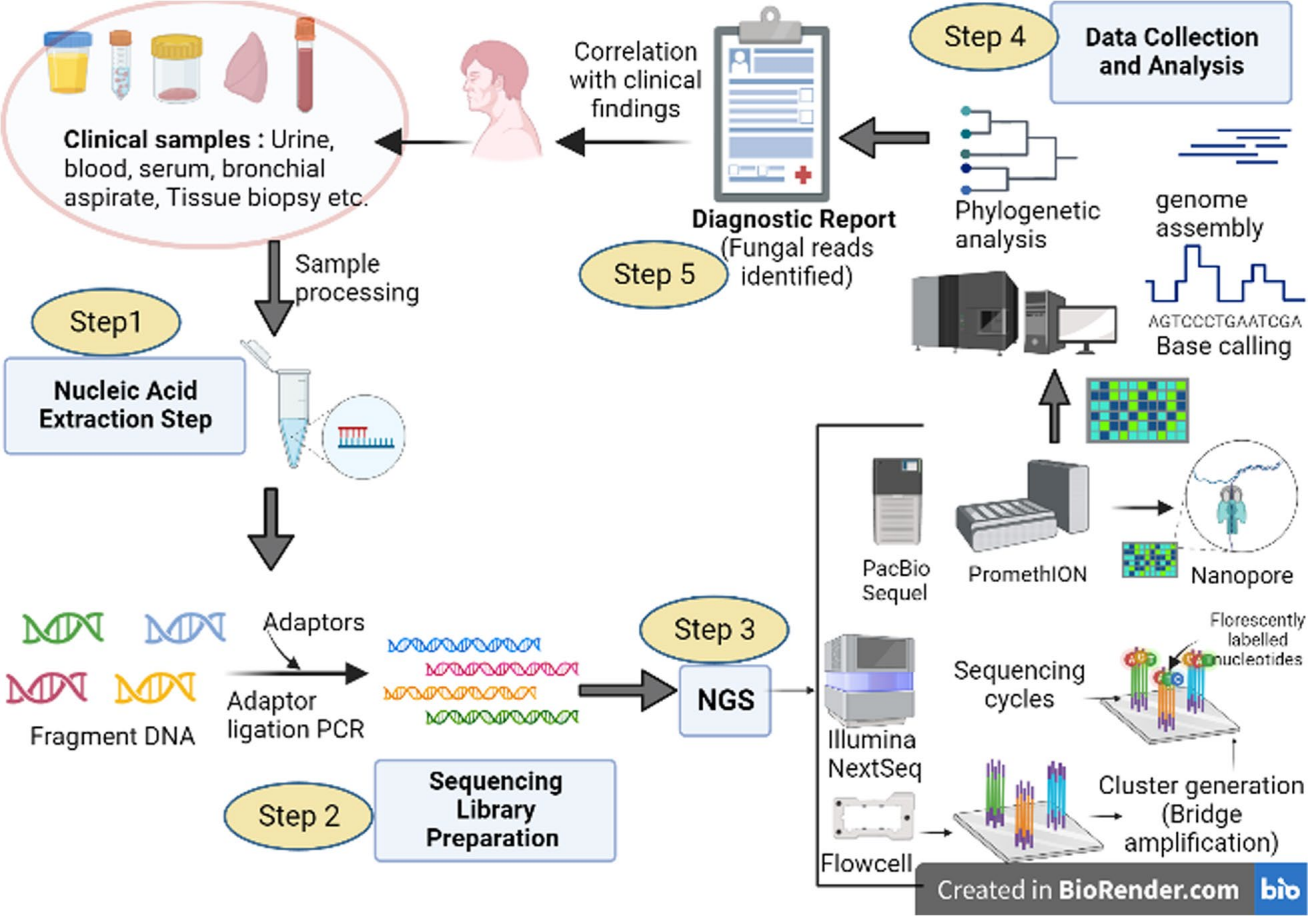
Schematic diagram depicting workflow of NGS for application in fungal pathogen diagnosis. [The figure has been created in Biorender.com]

In principle, the concepts behind Sanger and next-generation sequencing (NGS) technologies are similar. But, NGS, also referred to as “high-throughput sequencing” (HTS), is massively parallel, offering the power of sequencing hundreds to thousands of genes or gene regions simultaneously at one time. By identifying clinical samples’ nucleotides and comparing them to a catalogue library [168, 169]. Because of its ability to precisely and rapidly screen for multiple gene targets closely related to pathogens [170, 171], NGS is unquestionably ushering in the new era of precision medicine. A schematic diagram showing the workflow of NGS has been depicted in Fig. 3.

In the last two decades, multiple new high-throughput sequencing (HTS) techniques and new sequencing platforms have revamped the stage of NGS. These HTS include the next-generation “short-read” and third-generation “long-read” sequencing methods. The short-read technologies have emerged since 1994 and were commercialised in 2005. This uses miniaturised and parallelized platforms for the sequencing of 1 million to 43 billion short reads (50–400 bases each) per instrument run. These include the powerful benchtop sequencers, i.e., Illumina MiSeq, Illumina HiSeq, Ion Torrent: Proton/PGM sequencing, SOLiD, cPAL etc. The Illumina NextSeq500 is capable of giving massive data by sequencing 30–40 fungal genomes in a single run [172]. However, for reading a long stretch via the first-generation sequencers, the strands must be fragmented and amplified, and computer programmes are then used to assemble these random clones into a continuous sequence [173]. To overcome this challenge, the third generation “long read” sequencing methods came into light. Long-read sequencing technologies are capable of reading longer lengths of between 5000 and 30,000 base pairs. This eliminates amplification bias and thus generates a reasonable length to overlap a sequence for better sequence assembly [174]. The Single molecule real-time (SMRT) sequencer (developed by Pacific Biosciences) can sequence lengths exceeding 10,000 bases in less than two hours. In a SMRT sequencer, DNA polymerase is placed at the bottom of a zero-mode waveguide chip. This chip is a nanostructure fabricated in a thin metallic film capable of confining an excitation volume to the range of attoliters. A circular single-stranded structure made of two adapters is formed by the DNA molecule inside each zero-mode waveguide chip. DNA polymerase is used to sequence a pair of complementary strands, and the nucleotides are found by measuring the fluorescence [175]. Next is the Oxford Nanopore sequencer. This is a unique, scalable technology that enables direct, real-time analysis of long DNA or RNA fragments. Its working principle is based on monitoring the changes in an electrical current as nucleic acids are passed through a protein nanopore. The resulting signal is then decoded, giving the specific DNA or RNA sequence [176, 177].

Metagenomics NGS (mNGS) is a powerful platform in the field of clinical mycological diagnostics. The first case wherein NGS was used was in a 61-year-old patient presenting symptoms of eosinophilic pneumonia accompanied by bronchial asthma. Direct analysis of sputum samples by shotgun sequencing confirmed the co-infection with *Aspergillus fumigatus* and *Schizophyllum commune*. NGS has been successfully employed in the diagnosis of fungal pathogens and many of those have reported the presence of more than one fungal strain involved [177–183]

Of interest is the diagnosis of *Pneumocystis jirovecii*, which is an unculturable fungal pathogen and whose nonspecific clinical presentation makes its diagnosis challenging. Zhang et al. [184] reported 13 cases of Pneumocystis pneumonia (PCP) detected by shotgun metagenomics sequencing. All 13 enrolled patients had pneumonia of unknown etiology. 11 of the 13 cases had *Enterococcus* spp., Nocardia, Human herpes virus, *Candida* spp., and *Aspergillus* spp. infections, which were also detected by metagenomic NGS (mNGS). Further, in three cases where the patients were unable to tolerate bronchoscopy examinations or other invasive procedures, NGS was able to directly detect the pathogen in peripheral blood samples. This suggests that for seriously ill patients who cannot endure invasive procedures, high-throughput sequencing offers a reliable alternative. The inadequate sensitivity of conventional diagnostic platforms in diagnosing PCP has been shown by Chen and team [183]. The study reported the case of a 27-year-old woman with renal transplantation that developed pneumonia four months after her transplantation surgery. Blood tests revealed a low CD4 count. Microscopic examination of BAL and sputum did not show any fungal morphology. Also, serological and culture tests and blood culture tests were all negative. Serum galactomannan and BDG levels were both normal. On day 13th of hospitalization, diff-Quick staining for *Pneumocystis jirovecii* was still negative and the BALF culture revealed no growth of bacteria or fungi; the galactomannan level was in the normal range. Finally, 3 mL of BAL sample was sent to the MGISeq 2000 platform for NGS. Results revealed the presence of 10 bacterial species, one dominant fungal species, i.e., *Pneumocystis jirovecii*, and one viral species. This confirmation led to the initiation of the correct treatment regimen with effective resolution of the patient. Based on the results, the team has advocated the need to add PCP prophylaxis to the Diagnosis and Treatment Guideline of IFIs in Solid Organ Transplant Recipients in China. There have been other studies as well that have reported the successful use of mNGS to diagnose pulmonary infections involving *P. jirovecii* [177, 185–187], highlighting the inadequate sensitivity of microscopic examination of samples and serum BDG assay for diagnosing PCP.

Similarly, *Cryptococcus* is another invasive fungal pathogen that has been successfully detected by NGS techniques as reported by past workers. Cryptococcosis is caused by two sibling species complexes, *Cryptococcus neoformans* and *Cryptococcus gattii*. Both these species have significant differences in their epidemiology, clinical manifestations, progression, and treatment strategies. *C. gattii* affects non-HIV patients with more serious neurological complications, poorer response to antifungal drugs, and a longer period of treatment required [188, 189]. At present, traditional laboratory diagnostic methods, including India ink staining, and cryptococcal antigen (CrAg) detection in CSF, cannot distinguish the two cryptococcal species. The use of dopamine containing media i.e. Banana blossom agar developed by Khayhan et al. [190] can help in enriching the growth of the complex and differentiate it from other yeasts but it cannot differentiate among the two. NGS is a technique that can easily help to distinguish the two species. This will aid in providing useful information on the species involved, essential for guiding correct decision-making.

In a comparison-based study, Xing and team [191] evaluated the diagnostic performance of mNGS with conventional Indian Ink staining and culture assay on CSF samples from 12 non-HIV-infected patients with cryptococcal meningitis (CM). Although Indian ink and culture of the CSF were positive for *Cryptococcus* in 83.333% (10/12) of the samples, and the mNGS was able to detect DNA in 75.3% of samples (9/12). However, the DNA of both *C. neoformans* s.l. and *C. gattii* s.l. was detected concurrently in 33.333% (4/12). The ability to distinguish between species is an additional benefit offered by NGS, thus informing the choice of appropriate antifungal treatment courses. In a similar recent study, the diagnostic performance of NGS was tested on 197 non-HIV patients with suspected central nervous system infections [192]. Of the 197 cases, 46 were confirmed as cases of CM. Further, 43 cases were of *Cryptococcus neoformans* infections and 3 were of *Cryptococcus gattii* infections. The sensitivity and specificity were 93.5% and 96.0%, which was higher than that of the Indian Ink and culture tests but slightly lower than for CrAg detection by ELISA. Noteworthy is that it was only mNGS that could identify *Cryptococcus* at species level. CSF mNGS can be considered as a supplementary test to diagnose CM, but further research on improving the sensitivity may focus on the removal of human sequences and possible pathogen sequence enrichment. In addition to detecting CM, Zhang et al. [180] reported that mNGS was also able to detect a rare case of Cryptococcal osteomyelitis in two non-HIV patients that were suspected to have soft tissue neoplasm. With microbiological culture being negative, mNGS was able to identify *Cryptococcus neoformans* and, with oral fluconazole-only treatment, the infections were successfully eradicated.

NGS holds the potential to discover rare fungal pathogens, especially those that cannot be readily isolated or those that have a very low incidence in non-endemic areas. Such cases have a higher chance of being misdiagnosed or underdiagnosed by the physician. For example, histoplasmosis is an endemic disease mainly occurring in North America and is rare in China. In one such study, a 27-year-old Chinese man presented with chronic pulmonary lesions for more than a year, followed by lesions reaching the epiglottis that led to progressive pharyngeal pain. After a series of tests, the patients were diagnosed as having acute epiglottitis and suspected tuberculosis (TB). But, with worsening of symptoms, finally mNGS analysis of the epiglottis tissue and BAL were performed, which identified *H. capsulatum* within 96 h. The patients received itraconazole for 12 weeks, and post-treatment, chest CT showed complete regression of the lesions and effective resolution of infection. Thus, although the incidence of laryngeal histoplasmosis is rare, mNGS should still be exploited to attain a precise diagnosis in cases where persistent ground-glass density shadows continue to appear in imaging scans with worsening of symptoms. Also, the patient’s clinical history with acquired immunodeficiency or any inborn errors of immunity may also be taken into consideration. Moreover, NGS can also be a useful monitoring tool for studying disease progression and assessing the effectiveness of antifungal therapy, as NGS done during consecutive follow-up visits can be analysed by comparing the abundance of sequence reads with the clinical recovery of the patient [193].

With the ability to diagnose rare, atypical cases, samples with low fungal loads and for simultaneous detection of multiple etiological agents from different taxa (bacteria, viruses, and fungal pathogens) in a single sample (in cases of mixed infections), NGS represents a useful tool in the diagnostic armamentarium. However, it suffers from challenges hindering the routine implementation of NGS technology in IFIs' diagnosis plans, and at the moment, the high cost is the major obstacle that needs to be reduced for wider acceptability [194]. Also, the analysis of NGS data for multiple pathogen identification using the nucleotide sequence databases as reference data requires larger memory size computer systems, and day-to-day NGS analysis by clinical laboratories would definitely require major improvements in data processing capabilities [195]. As of right now, NGS can be used as an extra diagnostic tool, and the results need to be compared to the results of traditional diagnostic tests and the patient’s condition.

### Advances in biosensor-based fungal diagnostic tests

According to the International Union of Pure and Applied Chemistry (IUPAC), biosensors are defined as “integrated receptor-transducer devices that provide quantitative or semi-quantitative analytical information using a biological recognition element” [196]. A biosensor has three main components on which they work: (a) a recognition element that distinguishes a particular analyte or a group of analytes; (b) a transducer element that produces a signal; and (c) the signal processor. They function when the biosensor's recognition and transducer components cooperate to provide a quantifiable signal. Biosensors have revolutionised healthcare and diagnostics since their development. Biosensor technology can be used to create inexpensive, disposable, point-of-care gadgets or to provide continuous monitoring of different biomarkers in the least invasive way possible [197, 198]. The advancements in biosensor technology, which have already and will continue to do so, have also benefited invasive fungal diagnostics, as this section has highlighted. Depending on the type of the signal generation system or transducer, biosensors are classified as: (i) electrochemical, (ii) optical, (iii) piezoelectric, or (iv) thermometric. We will be discussing the new advances in the various types used in the identification of invasive fungal species.

*Electrochemical biosensors* electrochemical biosensors analyse the content of a biological sample based on the direct conversion of a biological reaction into a measurable electronic signal, e.g. changes in impedance (electrochemical impedance spectroscopy), changes in current (amperometric) or potential difference (potentiometric) [199]. But so far, only a small number of publications have talked about how they can be used to diagnose fungi. To directly detect *C.albicans*, Kwansy et al. [200] created a straightforward electrochemical impedance-based biosensor. The biosensor was able to directly capture yeast cells on electrodes specifically functionalized with anti-Candida antibodies, causing changes in the electrochemical impedance spectroscopy. The advantage of using such a technique is that the biosensor can detect yeast cells in the test specimen in less than an hour, saving the time to decide on the antifungal therapy. The transfer charge resistance increased with increasing yeast cell concentration (i.e. Rct values of 350, 500, and 578 Ohms for 10, 100, and 1000 CFU/mL, respectively) with a linearity fit of R^2^ = 0.916 and a demonstrated sensitivity of capturing as low as 10 CFU/mL in PBS sample. The team is working towards further refining and testing the biosensor in clinical samples. In a different work, a team described the creation of an electrochemical biosensor for detecting the pathogenic glip target gene, which can be used to diagnose IA [201]. The glip gene codes for a fungal toxin (i.e., gliotoxin) found exclusively in *Aspergillus* species. The 1,6-hexanedithiol self-assembled monolayers produced on the gold electrode were used to immobilise the glip-probe onto chitosan stabilised gold nanoparticles (AuNPs) for the electrochemical biosensor. Based on the hybridization reaction and the signal produced using toluidine blue as an indicator molecule, the sensor's capacity to detect glip-T was examined. In standard buffer and real sample, the detection limit of the glip biosensor was 0.32 0.01 × 10–14 M and 0.81 0.01 × 10–14 M, respectively. The developed biosensor was able to detect glip-T in merely 20 min, offering fast detection of IA-causing and gliotoxin-producing strains of *Aspergillus fumigatus*. Additionally, the technology demonstrated strong reusability, making it a financially advantageous choice for its conversion into a handheld compact device for onsite glip-T detection of patients with IA.

*Optical biosensors* These particular biosensors are the most popular and frequently used of all. An optical biosensor consists of a biorecognition sensing component combined with an optical transducer system. The biosensor is compatible with a range of biomolecules (enzymes, antibodies, antigens, receptors, nucleic acids, and whole cells as the biorecognition elements). The transducer system can be based on surface plasmon resonance (SPR) or evanescent wave fluorescence, or dynamic light scattering, refractometry, etc. [202]. These offer advantages over other analytical methods since they enable us to achieve label-free detection of the analyte as well as enable real-time observations. Cai and colleagues [203] used a photonic crystal (PC) based on protein-carbohydrate specific recognition to detect the presence of *C. albicans*. The analyte reaction was based on the specific interaction between Concanavalin A (Con A) and mannan present on the cell wall of fungi. For this, monodisperse 2-dimesional (2D) PC arrays of pure ConA protein hydrogels were created by crosslinking Con A solutions with glutaraldehyde. Finally, a readable blue-shift of the 2D array diffraction that was proportional to the fungal load resulted from cross-linking that happened as a result of the hydrogel Con A proteins recognising multiple mannose groups. This shrinking and decrease in the 2D array particle spacing was the final step. The sensor had a good detection limit of 32 CFU/mL for *C. albicans* and was able to distinguish specifically between *C. albicans* cells and other microorganisms. In order to increase the sensitivity and shorten the detection periods, the team has been further optimising and working on employing less crosslinked, thinner hydrogels. Nonetheless, this study provides proof of concept that interactions based on lectins and carbohydrate antigens can form the basis of designing potent biosensors that would be rapid, easy to use, and economical at the same time. Besides ConA, there is another c-type lectin, i.e. dendritic cell-associated lectin-2 (dectin-2) that is known to bind fungal mannan [203]. Dectin-2 binds to high-mannose structures and its binding leads to a series of inflammatory responses. The ability to exploit the properties of dectin-2 in the development of dectin-based biosensors is another possibility [204].

In a recent 2022 study, a team of scientists reported the development of a novel optical nano-biosensor based on the dynamic light scattering (DLS) method for the diagnosis of IA based on the detection of *Aspergillus* galactomannan in biological fluids suitable for express POC diagnostics. Such biosensors determine the presence of an analyte in solution by the detection of the aggregation of nanoparticles functionalized with receptors for the target analyte. In this study by [205], gold nanoparticles were functionalized with high-affinity antibodies to galactomannan (GM) as these were used as probes. The hydrodynamic diameter of functionalized nanoparticles as well as the count rate of scattered light pulses were used by the researchers as two separate analytical signals. Clearly, this was done to improve the precision and dependability of dynamic light scattering (DLS)-based nanosensors. The data for the hydrodynamic diameter and the count rate were compared, and this revealed a good correlation between the two. When measuring with clinical BAL samples, the coefficient of determination between the values of the hydrodynamic diameter and absorbance of ELISA was R^2^ = 0.9705, and between the values of the count rate and absorbance, it was R^2^ = 0.998, showing good correlation between biosensor and ELISA. The detection limits for the evaluation of GM calibrator in plasma were 0.2 ng/mL as per hydrodynamic diameter data and 0.1 ng/mL as per the count rate data. The detection limit for GM in BAL samples was 3 ng/mL when detected by the hydrodynamic diameter and 0.4 ng/mL when detected by the counting rate of scattered light pulses. With comparable proficiency as ELISA, the biggest advantage is the rapidity, as this washing-free technique was able to process and give measurement of 10 samples in less than 100 min. The results warrant future exploration of the proposed method for designing express point-of-care diagnostics.

In addition to the above biosensors, piezoelectric biosensors and thermal biosensors also hold great potential for being exploited for fungal detection. A piezoelectric biosensor has a piezoelectric crystal. The oscillations of the piezoelectric crystal surface vary when the analyte binds to it [206, 207]. The analytical signal is measured using this shift (decay) in oscillatory frequency, which is proportional to the mass bound to the crystal. Real-time label-free operation and fungal growth monitoring may be possible using mass detection [208]. Similar to that, thermal biosensors monitor the change in heat that results from a biological reaction in a medium [204, 209]. Piezoelectric or thermal biosensors have not yet been reported to be used for the detection of invasive fungus species. In a 2005 study, Nugaeva and colleagues [210] created a micromechanical cantilever biosensor for the rapid quantitative detection of *Saccharomyces cerevisae* and *Aspergillus niger*. Needless to say, more research needs to go into exploring these types of biosensors in the field of fungal pathogen detection to establish proof of concept. In addition to the above, there have been past studies focusing on nano-size materials (such as carbon dots, carbon nanotubes, nanowires, liposomes etc.) that can be incorporated into the designing of novel and unique biosensors, enabling higher precision and faster detection of invasive fungal strains [211–213] as shown in Table 2.

**Table 2:** Nano-size materials used in fungal detection

Fungal strain	Material	Methodology used	Outcome	References
*C. albicans*	Carbon Nanotubes	• Developed biosensor based on a carbon nanotube field-effect transistor (FET) to detect pathogenic yeasts even at low concentrations • Monoclonal anti-Candida antibodies were adsorbed onto the single wall carbon nanotube (SWCNT) to provide specific binding sites for fungal antigens • Upon interaction between antibody and antigen, the FET device displays change in electrical current	• FET device displayed stable sensor response for more than 10 days • Able to detect as low as 50 CFU/mL of *C. albicans* and that too only in 1 h	Villamizar et al. [211]
*Phytophthora cactorum*	Nanoparticles of TiO_2_ or SnO_2_	• Nanoparticles of TiO_2_ or SnO_2_ on screen-printed carbon (SP) electrodes were fabricated • These metal oxide nanoparticle-modified electrodes were used for amperometric detection of a volatile compound *p*-ethyl guaiacol indicating presence of Phytophthora	• Metal oxide nanoparticle modified electrodes showed high sensitivity and low detection limit (35–62 nM) for the detection of *p*-ethyl guaiacol along with high repeatability	Fang et al. [222]
*Mucor circinelloides*	Nanocoating with gold nanoparticles (AuNPs)	• Presented a novel concept of cell nanocoating • Use of specific markers (fungal or bacterial) to induce nanocoating of AuNPs based on reduction of disulfide bonds • Induction of plasmonic AuNPs nanocoating after interaction with cell surface markers (using surface molecules, including disulfide- bond-containing (Dsbc) proteins and chitin) upon addition of reducing agent • Use of plasmonics and fluorescence as transduction methods	• Rapid microbial screening using specific cell nanocoating by targeting surface molecules on the microbial surface • Detection in a short time (5–30 min) • Detection can be performed with the naked eye or using a hand-held fluorometer (limit of detection was 35–1500 CFU/mL)	Xu et al. [223]
*A. niger*, *Aspergillus oryzae*, *Penicillium chrysogenum* and *Mucor hiemalis*	Plasmonic AuNPs	• Developed a protocol to formulate AuNPs that upon reaction with specific spore forming fungi, causing changes in shape and morphology of AUNPs resulting in visible changes in color	• High sensitivity (80%) and 95% specificity with detection limit of 10 CFU/mL • Easy and simple readout (color change) with naked eye • Useful for rapid detection of fungal spores for hygiene control and self-diagnosis	Sojinrin et al. [224]
*Aspergillus niger, Penicillium chrysogenum, Alternaria alternata*	Fluorescent Carbon-Dots thin film	• Developed a novel method i.e. CDs-based thin film as a sensor for detection of fungal spores • The thin film of carbon dots deposited on quartz plates was achieved using the Blodgett technique • To test CDs in the thin film form as a sensor, 12 thin films were arranged in the enclosed box at different locations and *A. niger* the repetitive model fungus • The interaction between CDs and *A. niger* fungus caused changes in the fluorescence emission properties of CDs that was captured	• Easy fabrication, low cost, high stability, economical for rapid detection of fungal growth and spores	Gaikwad et al. [213]
*C. albicans*, *C. tropicalis*, and *C. krusei*	Magnetic nanoparticles	• Developed a protocol for direct identification of Candida from serum • For this, magnetic microspheres (Fe_3_O_4_) were modified by polyethylenimine (PEI) to form Fe_3_O_4_@PEI • The team then prepared positively charged silver nanoparticles (AgNPs) as the substrate for surface-enhanced Raman scattering (SERS) • *Candida* was directly identified from serum by SERS detection	• Direct rapid and non-destructive detection of *Candida* under non-culture conditions from serum sample • Test completed within 40 min) • Test accuracy close to 99.8%	Hu et al. [225]
*A. niger*	Peptide modified AuNPs	• *A. niger* spore-binding peptide ligand identified by phage display screening • AuNPs modified with a specific binding peptide • Peptide modified AuNPs when bind to *A. niger* spores show aggregation and a rapid visible change in the color	Rapid (< 10 min) sensitive (as low as 50 spores) detection of fungal spores	Lee et al. [226]

*Microfluidic-based detection* Microfluidic-based methods are gaining importance in the field of fungal diagnostics [214, 215]. Microfluidics involves the processing of small quantities of fluids by using tiny forces at the microscale dimension. This technique relies on precise control and manipulation of fluids that are geometrically constrained to a small scale. The fluid properties change and surface forces dominate volumetric forces at this scale. The microfluidic chip is a pattern of microchannels that has been moulded or engraved. A number of holes of various sizes that are bored out across the microfluidic chip connect this network of microchannels to the macroenvironment. Faster reaction times, better temperature control, portability, the integration of lab procedures into one device (lab-on-a-chip), easier automation and computerization to attain high precision analytical capabilities make this technology worth exploring [216–218]. Looking into the role of microfluidic chips in the detection of fungal pathogens, Asghar and team [219] have contributed in this direction. They reported designing an innovative immuno-based microfluidic device for rapid detection of *C. albicans* from phosphate-buffered saline (PBS) and whole human blood. The microfluidic chip is made of poly (methyl methacrylate) (PMMA), double-sided adhesive (DSA), and a glass cover that was precisely laser-cut to contain three microfluidic channels, inlets, and outlets. Anti-Candida antibodies were immobilised on the surface via surface chemistry based on protein G. The team then used monoclonal and polyclonal antibodies to evaluate the efficiency of capturing *C. albicans* from spiked samples inside microfluidic channels. Polyclonal anti-Candida antibodies showed higher values (77.4 ± 4.4%) than monoclonal anti-Candida antibodies (48.6 ± 2.8%). In spiked PBS samples, there was an increase in capture efficacy with increasing fungal load, i.e., with increasing load from 10^3^, 10^4^, and 10^5^ CFU/mL, the capture efficiencies also increased to 61 ± 12.7%, 70 ± 13.2% and 77.4 ± 4.4%, respectively. At 10^2^ CFU/mL, the capture efficacy initially decreased, but the team found that by increasing the sample volume (from 50 µL to 1 mL), high capture efficiencies were observed for 10^2^ CFU/mL (78 ± 13.2%) and 10 CFU/mL (75 ± 21.1%). For spiked whole blood, the capture efficiency was 40.5 ± 4.7%, possibly due to the large number of blood cells hindering the Candida–antibody interaction. Hence, the team lysed the spiked blood sample as a result of which the capture efficiency significantly increased to 74.6 ± 6.8%. The whole process took an average of 1–5 to 2 h. Besides this, the microfluidic approach allows the capture and isolation of whole Candida cells (as Candia are not lysed in the protocol), so drug resistance and susceptibility testing may be possible. Further, microchip techniques can be integrated with smartphone-based imaging [220] to enable point-of-care testing as well as remote patient testing and care. On similar lines, Bras et al. [221] also studied the use of a microfluidic based approach for multiplexed, point-of-need detection of fungal plant pathogens infecting grape cultivars.

The data reviewed on use of biosensor technology in this section emphasises the fact that biosensor based fungal diagnostics represents a new era worth exploring and holds immense potential for changing the face of fungal diagnostics. By combining and integrating different and crucial recognition elements on a multiplexed biosensor platform, the challenges of sensitivity, specificity, and reproducibility can be overcome. Biosensors can speed up analysis, shorten sample preparation times, and offer a low-cost, straightforward diagnostic tool to record the data. This makes it possible to perform point-of-care (POC) diagnostics using biosensors. They can be placed as first line soldiers (as part of routine screening) in the diagnostic regimen, eliminating the need to run every suspicious test sample and perform rounds of long, laborious and expensive tests (culture tests, PCR, immunoassays, ELISA, etc.). Advances in biosensor research point-of-care testing and real-time monitoring of fungal markers and fungal growth would aid in timely detection of the fungal pathogen, giving buffering time to start the most appropriate intervention course at the earliest.

### Artificial intelligence and machine learning: a new era in fungal diagnostics and patient care

Artificial intelligence (AI) is the simulation of human intelligence processes by machines, especially computer systems, and this simulation includes learning, reasoning, and self-correction. Machine learning (ML) is a branch of AI and computer science that focuses on the use of data and algorithms, allowing the software application to become more accurate at predicting outcomes just like humans do. ML algorithms use historical data as input to predict new output values more accurately. Deep learning is further defined as a subset of machine learning techniques that teach computers to do what comes naturally to humans, i.e., learn by example and from large datasets [227].

Over the last decade, AI methods from machine learning to deep learning have contributed immensely to accelerating digitalization in healthcare. Digitized healthcare offers fewer human errors, thus improving clinical outcomes and allowing real-time tracking of patient data etc. [228, 229]. The biggest advantage offered by AI and ML-based models is that they assist physicians in faster and more accurate decision-making by rapid processing and analysis of massive and complex data. A doctor relies on computed tomography (CT) scans or magnetic resonance imaging for later analysis, whereas an AI model would do the same job and spot the diseased area’s features in a fraction of a second. AI and machine learning have emerged as powerful tools for assisting diagnosis and providing a more precise diagnosis, aiding the physician in correct decision making. AI-based detection also enables identifying patients with under-diagnosed, latent or subclinical infectious diseases. There has been a study showing several cases with the emergence of cutaneous deep-seated fungal infection, months or years after transplantation at the healed sites, suggesting persistence of fungal organisms in a latent state [230]. This can be the case when fungi remain overt and flare up only when neutropenia and immunosuppressive medications impair immune defences sufficiently to allow reactivation and fungal proliferation. With AI models for disease detection, there is an ample opportunity to drive earlier diagnosis for patients in need, thus guiding correct treatment earlier in their disease journey [231]. In this section, we detail and improve our understanding of the use of AI and ML-based models in aiding accurate and faster detection of IFIs. We have continued to use simple terminology, keeping in view that readers are not from core computer science backgrounds. However, a detailed discussion of the various algorithms used, neuronal networks and their working or mode of operation, image analysis and processing principles, and classifiers used for data interpretation have not been covered because it is beyond the scope of this review.

*A basic overview of how ML works* In the simplest way, classical machine learning uses algorithms (a step-wise method to solve a problem) to become more accurate in its predictions. These ML algorithms are able to process large amounts of data and extract useful information. They keep on improving upon their previous iterations by learning from the data they are provided [227, 232]. There are four basic approaches: supervised learning, unsupervised learning, semi-supervised learning, and reinforcement learning. In supervised learning (task-driven), which is the most basic form of ML, the data scientist provides the algorithms with labelled training data and a smaller data set to train on. The dataset has already defined variables that the algorithm has to assess for correlations. Both the input and the output of the algorithm are specified. At the end of the training, the algorithm has an idea of how the data works and the relationship between the input and the output. The algorithm is then tested on the actual or final dataset. These algorithms keep on improving after being deployed, discovering new patterns and relationships as they train themselves on new data [233, 234].

**Fig. 4:**
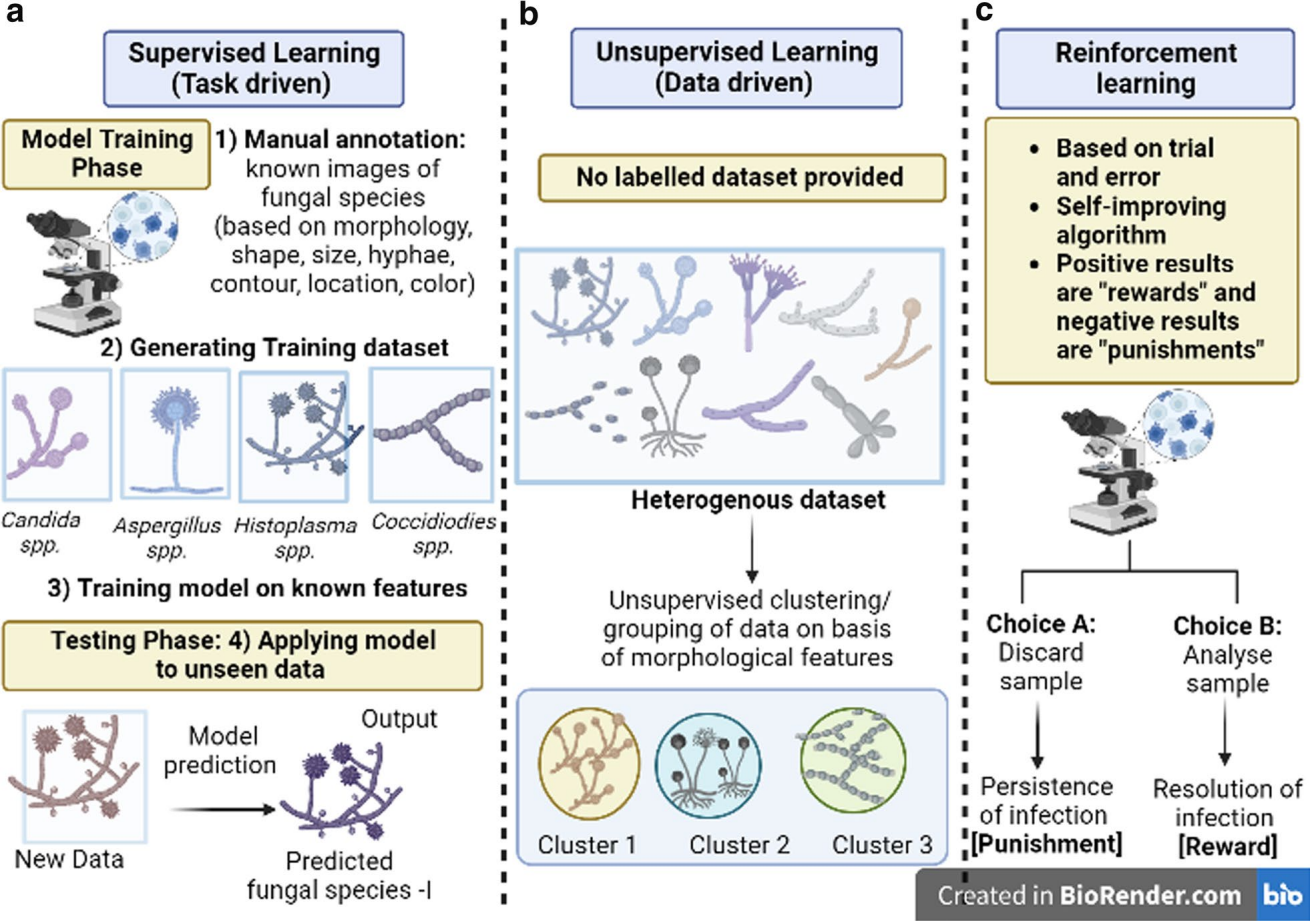
Schematic diagram of the three basic machine-learning paradigms: **a** Supervised learning, **b** Unsupervised learning and **c** Reinforcement learning explained in terms of fungal species detection based on microscopic images as datasets [The figure has been created in Biorender.com]

The unsupervised learning (data-driven) approach uses algorithms that are trained on unlabeled data. The algorithm searches through data sets in search of any significant connections. The benefit of supervised machine learning is its capacity to operate on unlabeled data. This means that no human intervention is needed to make the dataset machine-readable, enabling the programme to function on much larger datasets. The third category, i.e., semi-supervised, is a mix of the first two types. Data scientists may provide an algorithm with mostly labelled training data, but the algorithm may also independently examine the data and come to its own conclusions about the data set. Finally, reinforcement learning (learning from error) directly mimics how people learn from data in their daily lives. It has a self-improving algorithm that adapts to new circumstances and learns from mistakes. [233–235]. Positive results are “reinforced” or encouraged, while negative results are “punished” or discouraged. The same has been explained in Fig. 4.

The basic steps involved in any ML-based model include:

- Step 1: We need to figure out what the problem is and what we want to achieve.
- Step 2: We must collect raw data, also known as the data acquisition step.
- Step 3: We must clean/manipulate the data (for outliers, null values, error correction, and so on), in order to use it in our machine learning training. Furthermore, we will split it into a training dataset and a final testing dataset.
- Step 4: We need to choose the algorithm.
- Step 5: We need to train the chosen algorithm (model-training) using training datasets to improve our model’s ability to predict the output as desired.
- Step 6: We need to test or evaluate the algorithm on a testing dataset (model-testing) to get the model’s performance. This metric allows us to see how the model might perform against data that it has not yet seen.
- Step 7: Fine tuning of the algorithm to improve on performance parameters.

A few terms used in the cited studies have been defined for clearer understanding, and these include:

- Artificial neural network (ANN): This is a computational model that consists of several processing elements that receive inputs and deliver outputs based on their predefined activation functions. This is inspired by the biological neural networks of human brains.
- Convolutional neural network (CNN): Because of its ability to recognise patterns in images, CNN is a type of artificial neural network used primarily for image recognition and processing.
- Deep neuronal networks (DNN): this is a type of artificial neural network with some level of complexity, usually at least two hidden layers between the input and output layers.
- Support Vector Machines (SVM) are supervised learning algorithms that analyse data for classification and regression.
- Natural language processing (NLP): it is a subfield of artificial intelligence concerned with the interactions between computers and human language, i.e. how computer programmes process and analyse large amounts of natural language data.
- Bag of words: It is a simplified representation used in natural language processing and information retrieval (IR). In this, a text (sentence or document) is represented as a bag (multiset) of its words, disregarding grammar or word order.
- A classifier in machine learning: it’s a type of machine learning algorithm used to map the input data to a specific category, i.e. assign a class label to a data input.
- A classification model tries to draw some conclusion from the input values given for training. It will predict the class labels and categories for the new data.
- ImageNet is the largest visual database collection designed for use in visual object recognition software research with more than 14 million images hand-annotated.

Many AI and ML-based models have been explored to assist at many levels in fungal diagnostics. This includes: (a) assistance in microscopic image analysis for precise detection; (b) assistance in histopathological slide analysis (c) assistance with medical imaging scans (X-rays, MRI, CT, and so on) and (d) others. We will be discussing all of these with proof of concept from recent studies.

*Assistance via automated microscopic image analysis* Initial diagnosis of fungal infections still relies on microscopic examination of fungi or fungal structures. However, in many cases, it does not allow unambiguous identification of the species due to visual similarities. Additional biochemical tests or molecular approaches, such as PCR or sequencing, may be required, adding to the overall cost and time. In the future, AI-assisted microscopy systems will be on the front line of monitoring and investigating microorganisms. Zielinski et al. [236] presented a novel concept of using deep neural networks to classify microscopic images of various fungi species. The multi-step algorithm was used to produce robust image features using previously trained DNN, aggregate them using the bag-of-words approach, and classify them with SVM. The approach was able to identify morphologically similar looking species (or misclassified species, especially those belonging to the genus Candida, *Cryptococcus*, and Saccharomyces) based on set visual parameters trained for the classifier, which included brightness, size, shape, arrangement, color, quantity, shape, etc. Such an approach would definitely eliminate the last stage of biochemical identification, thus shortening the identification process by 2–3 days and accelerating the next course of action by the physician, saving time and improving patient care. On similar grounds, Lv et al. [237] developed an intelligent system based on a deep learning algorithm for automatically diagnosing fungal keratitis based on in vivo confocal microscopy (IVCM) images. The model included 2088 IVCM images in the training dataset (consisting of images with and without fungal hyphae) and a total of 535 images in the testing dataset. In the testing dataset, 515 images were diagnosed correctly and 20 were misdiagnosed, giving an accuracy of 96% in detecting fungal hyphae. Such deep learning algorithms have the potential to change the current mode of disease diagnosis. These models definitely need to be tested and their applicability evaluated by performing on a larger database of samples in multicentre research studies.

Koo and colleagues [238] developed a deep learning model for automatic detection of hyphal structures with the advantages of having a quicker, convenient, consistent, and automated technique. To develop this autodetection model, the team first generated training datasets using recorded video of images processed by KOH staining. Dataset-100, Dataset-40, and Dataset-all with the captured microscopic images of 100, 40, and both 100 and 40, respectively, were generated. The recorded video was converted into images frame by frame and the fungal hyphae location was annotated. The YOLO-v4 Network (with muti-Graphic Processing Unit deep learning server) was then trained using the training datasets followed by slide image evaluation on validation and testing datasets. For final detection and interpretation, two approaches were designed: image classification and object determination. If the microscopy image contains hyphae, the image classification system returns a positive; if not, it returns a negative. In the object detection approach, the system finds hyphae-like objects and evaluates the similarity of the found objects with previous trained datasets. The object detection approach is more sophisticated and gives detailed information about the location and size of the hyphae-like object present. The overall sensitivity and specificity in the combined (100 + 40) data model was 93.2% and 89%, respectively, indicating that the deep learning autodetection hyphal model was highly sensitive and specific and hyphae could be rapidly detected with reliable accuracy. Such an approach can be useful in the case of large batch sizes of microscopic samples for testing. Also, detection of superficial fungi on skin and skin scrapings, nail tissue, etc. in immunocompromised populations or transplantation candidates may be an early warning sign [239, 240]. Routine screening with such quick AI-based models may aid the physician in the early treatment of such skin infections that would otherwise easily find their way to deeper layers (due to a weakened immune system), eventually becoming an IFI. In the future, AI-assisted microscopy systems will be on the front line of monitoring and investigating microorganisms.

*Assistance via automated histopathology analysis* histopathology of tissue specimens is an essential diagnostic tool. But, screening slides for individual fungi to diagnose fungal strains can be time-consuming for pathologists, and sensitivity always remains a concern. Ai-assisted systems have been developed to assist in the rapid screening of histopathology slides [241, 242]. Neural networks are composed of layers of processing units called neurons that perform mathematical calculations. A CNN is specifically used for image processing, but it requires a large amount of data for training, which is not always readily available, especially in the medical field. Thus, U-NET-based segmentation networks (U-NETs) have been reported to be successfully used in digital histopathology. U-NET is a subcategory of CNN specifically developed for biomedical image segmentation with 23 convolutional layers, and it can work on fewer training images and yield more precise segmentations [243]. By analysing digitised histologic sections of human nail specimens, Jansen et al. [242] developed a U-NET based segmentation model for the precise detection of oncomycosis. For this, the team used a total of 644 histologic whole-slide images (WSIs) from four different laboratories, and these were digitized. The histology slides were then manually annotated. A U-NET based ML model was developed for predicting the presence of fungus for each pixel in a WSI with image segmentation for precise localization and was trained on the annotated WSIs. The model was able to detect 90.5% of WSIs with the fungi and showed a sensitivity value of 93% and a specificity value of 77%. The result of the ML model demonstrated good accuracy of 86.49%, which correlates well with the median accuracy of 87.84% achieved with the eleven board-certified dermatopathologists. Such AI-assisted models for analysing large histopathology slides may be applied to preselect possible slides with fungal elements from a larger batch, and further, the preselected ones may be re-evaluated and confirmed by a pathologist.

*Other ML models* In addition to the above, the use of artificial neuronal network-based models has been evaluated for accurate direct detection from human images as well as datasets. Black fungus is a rare pathogen that gained its popularity during the COVID-19 pandemic as it affected large sections of immunocompromised populations. The Mucormycetes of the order Mucorales, class Zygomycetes, are responsible for the fungus illness. Mucormycetes mould enters through the mouth, nose, or burned or damaged skin. The fungi can easily spread to the eyes, skin, lungs, and brain, causing vascular thrombosis that may lead to tissue necrosis [244, 245]. The mortality rates with involvement of the lungs and brain are high (over 60%) [246]. Henceforth, early detection is the key to success. Recent work by Karthikeyan and colleagues [247] focused on developing a hybrid learning-based neural network classifier (HLNNC) for rapid identification of black fungus. First, a unique custom dataset was created utilising images taken from COVID patients’ real-time records, and then the black fungus was added (along with images that included nonaffected cases). Using the HLNNC, the samples were trained on both normal and affected images. The HLNNC identified the black fungus based on image acquisition, pre-processing, feature extraction, and classification or categorization of images based on an object’s contours, pixel strength, and variation in image pixel intensities, etc. The proposed hybrid learning model offered the highest accuracy ratio of approximately 99.5%, advocating its future exploration for use in clinical settings. Such algorithms represent an easy-to-use and cost-friendly presumptive test option for the identification of black fungus disease before jumping into expensive investigations, such as MRI or CT.

There is another approach referred to as Electric Nose (e-Nose) that refers to an electronic sensing device intended to detect odours or flavours using sensor arrays and pattern recognition systems. E-nose is a rapid, non-invasive, online device comprised of an array of gas sensors and appropriate pattern recognition software [248]. Many fungi emit volatile compounds that form the basis for olfactory detection and identification of these organisms by electronic-nose (e-nose) [249]. By monitoring the resistance of non-specific gas sensors exposed to the odour, an e-Nose can detect and even distinguish between odours from two different fungus species. Currently, e-Nose technology has been used to detect *Aspergillus spp* [250], *Aspergillus fumigatus* and *Candida albicans* [251], *Fusarium* and *Rhizoctonia solani* [248].

In a study by Borowik et al. [248], the team integrated low-cost e-Nose technology with an ML model for the identification of two pathogenic fungi, i.e., *Fusarium oxysporum* and *Rhizoctonia solan*. Two construction models of e-Nose were designed using metal oxide gas sensors. A collection of features characterising the forms of the response curves were applied based on how the sensors responded to the presence of odours. Finally, two examined species of fungi were distinguished using a machine learning classification model developed and trained using the logistic regression method. Based on the volatile odours that fungal species emit, such e-nose integrated into various ML models can be used to detect and identify them.

The studies highlighted in this section emphasise the fact that AI and machine learning systems represent rapid and useful aids for the physician or researcher in determining the most effective method for detecting the fungal agent involved. AI, consisting of an array of algorithms, analytics, deep learning, and neural networks, is a field that is constantly expanding and yet much work is required to train the AI-based systems so as to enhance the prediction accuracy.

However, ML based models suffer from some major drawbacks. For example, in ML, the final algorithm is chosen based on accurate results after running the results on every possible algorithm. During the final training and testing of large amounts of data, errors are inevitable and at times removing errors becomes nearly a tedious task for the experts taking a lot of time to resolve. Further, choosing the correct algorithm is manual based and at times may take more time than expected. In addition to this, ethical concerns on trusting algorithms and the results is also a major issue. Since algorithms are developed by human, they are subject to bias at any level of development [252, 253]. No doctor can rely on these algorithms alone because the results of AI evaluations are only a possible diagnosis that needs to be checked by human intelligence.

## Conclusion

The review has enabled us to give insight into the advances and improvements made in diagnostics tools*, *i.e. the nonculture methods, to meet the changing face of diagnostics in both resource rich and resource limited settings. Timely diagnosis of IFI is necessary to prevent the high morbidity and mortality associated with systemic fungal infections, as late diagnosis always equates with a poor prognosis. This reason is sufficient why further studies, standardising the already developed technologies and deepening the knowledge of novel tools (such as novel PCR assays, new rapid POC assays, T2Candida, PCR/ESI–MS, machine or deep learning models, etc.) are all the more needed. We have a range of newer techniques that have recently surfaced and enjoy the benefits of being simpler to perform, with shorter turn-around time, and can be aptly applied for routine screening in high prevalence settings, thus serving best for POC testing. On the other hand, we have more complex and advanced assays developed that incorporate multiplexed systems with high precision and accuracy indices that could be restricted to cases with positive POC tests, keeping in mind the cost and sophistication involved. Increasing experience with PCR assays to directly detect fungi in clinical specimens, commercial PCR assays with improved performance and supporting clinical validation studies have permitted the inclusion of molecular tests into the second revision of the EORTC/MSGGERC for invasive fungal disease. However, for the incorporation and placement of the new assays developed into the diagnostic algorithms requires more validation data on larger patient cohorts through well-designed multicentre studies. Moreover, these new approaches have to be combined with the conventional assays, fitting them into the best possible location of a diagnostic plan suited to give the most reliable and accurate diagnosis.

## Data Availability

Not applicable.
